# MADS-Box Transcription Factor *VdMcm1* Regulates Conidiation, Microsclerotia Formation, Pathogenicity, and Secondary Metabolism of *Verticillium dahliae*

**DOI:** 10.3389/fmicb.2016.01192

**Published:** 2016-08-03

**Authors:** Dianguang Xiong, Yonglin Wang, Longyan Tian, Chengming Tian

**Affiliations:** The Key Laboratory for Silviculture and Conservation of Ministry of Education, College of Forestry, Beijing Forestry UniversityBeijing, China

**Keywords:** *Verticillium dahliae*, MADS-box transcription factor, microsclerotia formation, pathogenicity, secondary metabolism

## Abstract

*Verticillium dahliae*, a notorious phytopathogenic fungus, causes vascular wilt diseases in many plant species resulting in devastating yield losses worldwide. Due to its ability to colonize plant xylem and form microsclerotia, *V. dahliae* is highly persistent and difficult to control. In this study, we show that the MADS-box transcription factor *VdMcm1* is a key regulator of conidiation, microsclerotia formation, virulence, and secondary metabolism of *V. dahliae*. In addition, our findings suggest that *VdMcm1* is involved in cell wall integrity. Finally, comparative RNA-Seq analysis reveals 823 significantly downregulated genes in the *VdMcm1* deletion mutant, with diverse biological functions in transcriptional regulation, plant infection, cell adhesion, secondary metabolism, transmembrane transport activity, and cell secretion. When taken together, these data suggest that *VdMcm1* performs pleiotropic functions in *V. dahliae*.

## Introduction

*Verticillium dahliae* Kleb. is a devastating soil-borne plant pathogen that causes Verticillium wilt disease, which causes severe damages to diverse plant species worldwide, including economically important crops, ecologically significant trees, and shrubs (Klosterman et al., 2009; Wang et al., 2013). For instance, the smoke trees (*Cotinus coggygria* Scop.), an important colored-leaf tree species, present the famous red-leaf scenery of the Fragrant Hills Park in Beijing, China. However, since 2005, Verticillium wilt disease has caused serious mortality of the smoke trees and impacted the red-leaf landscape (Wang et al., 2013). *V. dahliae* infects the host plants through the roots and then colonizes and propagates in xylem vessel. Currently, there are no curative fungicides to control this pathogen and cure the infected plants. Melanized microsclerotia, dormant structures formed by *V. dahliae*, play crucial roles in disease spread and its long-term survival in nature (Green, 1980; Xiao et al., 1998). They germinate and enter the host when suitable hosts and favorable conditions are available (Wilhelm, 1955).

In order to better understand the molecular basis of pathogenesis and microsclerotia development in *V. dahliae*, many genes have been characterized over the past decades, especially genes involved in signal transduction pathways, such as *Fus3* ortholog *VMK1* (Rauyaree et al., 2005), MAPK *Hog1* (Wang et al., 2016), MAPK kinase *Msb2* (Tian et al., 2014), G protein β subunit (Tzima et al., 2012), small GTPase Rac1 (Tian et al., 2015), and cAMP-dependent protein kinase A (Tzima et al., 2010). However, transcription factors acting as integral components of these important signal transduction pathways are relatively less understood in *V. dahliae*. By contrast, functional analysis of transcription factors is much more advanced in other plant pathogenic fungi, i.e., in *Magnaporthe oryzae* (Lu et al., 2014; Kong et al., 2015; Tang et al., 2015).

The MADS-box transcription factors are highly conserved across fungi, plants, insects, amphibians, and mammals (Shore and Sharrocks, 1995). MADS is named from the initials of its four members: MCM1, AGAMOUS, DEFA, and Serum Response Factor (SRF; Schwarz-Sommer et al., 1990; Shore and Sharrocks, 1995). The MADS-box domain, usually located in the N-terminus, is a conserved region of about 56 amino acids that binds to the *cis* regulatory consensus sequence CC[T/C][A/T]_3_NN[A/G]G (Wynne and Treisman, 1992). Generally, there are two MADS-box transcription factor genes *Mcm1* and *Rlm1* in fungi that can be categorized into two classes: SRF-type and Myocyte Enhancer Factor 2-like (MEF2-like), respectively. However, two additional members *Arg80* (SRF-type) and *Smp1* (MEF2-like) have been found in *Saccharomyces cerevisiae* (Mead et al., 2002). The SRF-type MADS-box genes usually play important roles in diverse cellular processes including fungal growth, osmotic regulation, cell wall and membrane structure, mating type specificity, virulence, and primary and secondary metabolism, while the MEF2-like MADS-box genes play important roles in fungal pathogenicity. To date, the orthologs of *S. cerevisiae Mcm1* have been studied in only a few filamentous fungi, including *Sordaria macrospora* (Nolting and Pöggeler, 2006), *Sclerotinia sclerotiorum* (Qu et al., 2014), *M. oryzae* (Zhou et al., 2011), *Fusarium graminearum* (Yang et al., 2015), and *Fusarium verticillioides* (Ortiz and Shim, 2013). Taken together, fungal *Mcm1* orthologs are involved in vegetative growth, stress response, secondary metabolism, and virulence.

The availability of the complete genome sequence of *V. dahliae* provides a foundation for the study of the mechanisms of microsclerotia formation and pathogenicity (Klosterman et al., 2011; de Jonge et al., 2013). In this study, we addressed the roles of *Mcm1* ortholog (*VdMcm1*) in fungal development, microsclerotia and pathogenicity of *V. dahliae*.

## Materials and methods

### Sequence and phylogenetic analysis of MADS-box genes in *V. dahliae*

The MADS-box genes *VdMcm1* (VDAG_01770) and *VdRlm1* (VDAG_00939) were identified during homology search of the *V. dahliae* genome database (Broad Institute) using BLASTP program with *Mcm1* (CAA88409) and *Rlm1* (NP_015236) of *S. cerevisiae* as a query. The amino acid sequence alignments were performed with ClustalX2.0 (Larkin et al., 2007). The phylogenetic tree was constructed with MEGA 6.0 (Tamura et al., 2013) using the neighbor-joining method and the bootstrap test was replicated 1000 times.

### Strains and growth conditions of *V. dahliae*

The *V. dahliae* strain XS11 was used as the wild-type strain in this study (Wang et al., 2013). All strains were regularly cultured on potato dextrose agar (PDA) plates at room temperature. To test the growth rate and conidia production, the cultures were grown on PDA plates, and the experiment was repeated three times. Conidia were harvested from cultures grown in the fresh liquid complete medium (CM; Dobinson et al., 1997) by filtration through two layers of Miracloth (Calbiochem, Germany). The percentage of conidia with abnormal morphology was calculated by randomly recording 100 conidia, and the process was repeated five times. Mycelia were prepared in liquid CM and used for DNA and RNA extractions. For stress response assays, all the strains were grown on CM plates with Sorbitol, NaCl, or KCl. To analyze the cell wall properties, 10 μg/ml calcofluor white (CFW) was used to stain the germinated conidia and mycelia. To test surface hydrophobicity, 20 μl 0.12% bromophenol blue were placed on the surface of 16-day-old fungal colonies grown on PDA, and the colonies were observed after 1 h as previously described (Ruiz-Roldán et al., 2015). The 16-day-old fungal colonies grown on PDA were directly used for RNA isolation, and the obtained total RNA was further used for gene expression analysis. The microsclerotia was induced on a basal medium (BM) as described previously (Neumann and Dobinson, 2003; Xiong et al., 2014). In order to count the number of microsclerotia, a small part of cellulose (diameter 80 mm) was taken for microscopic examination and the relatively separated microsclerotium was regarded as single one. This experiment was repeated three times.

### Targeted gene knockout and complementation

*VdMcm1* deletion mutants were acquired with the split-marker method (Goswami, 2012). According to this described method, the upstream (~1.4 kb) and downstream (~1.1 kb) flanking sequences of *VdMcm1* were amplified with primers V1-F/V1-R and V1-1F/V1-1R, respectively (Table S3). The resulting upstream and downstream fragments were fused with a geneticin-resistant cassette with primers V1-F/Ge-R and Ge-F/V1-1R (Table S3), respectively, by overlap PCR. All the fragments were confirmed by sequencing analysis. The two overlapping fragments were directly transformed into the protoplasts of *V. dahliae* XS11. The transformants were selected on TB3 medium (Goswami, 2012) with 50 μg/ml geneticin. The successful replacement transformants were initially identified by PCR with primers V1-F/Ge-R, Ge-F/V1-1R, and M1-F/M1-R (Table S3). The Southern blot analysis was performed to confirm the homologous recombination event by using the DIG High Prime DNA Labeling and Detection Starter Kit I in accordance with the manufacturers' protocol (Roche, Germany). The probe fragment used for Southern blot was amplified from genomic DNA with primers Probefor and Proberev. The genomic DNA used for the Southern blot analysis was digested with *Kpn*I. For complementation, a fragment containing the native promoter (~2.0 kb) and the entire open reading frame of *VdMcm1* was amplified using primers N1-F/M1-R (Table S3). The resulting PCR products were co-transformed into protoplasts of Δ*VdMcm1-5* with hygromycin resistance cassette. Successful complementation was confirmed by reverse transcription PCR with primers RT-F/RT-R (Table S3).

In order to analyze the cellular location of VdMcm1, *VdMcm1-GFP* fusion was constructed by overlap PCR. A fragment containing a native promoter (~2.0 kb) and coding region of *VdMcm1* without stop codon was amplified using primers N1-F/Cd-R with Prime STAR HS DNA Polymerase [Takara Biotechnology (Dalian), China]. The GFP fragment was amplified from pKD5-GFP with primers GFP-F/GFP-R using Prime STAR HS DNA Polymerase. The resulting two fragments were fused together using overlap PCR with primers V1-F/GFP-R. The successfully fused fragment was co-transformed into protoplasts of strain *VdMcm1*-5 with hygromycin resistance cassette. Successfully integrated transformants (*Cp-Mcm1-GFP*) were selected with GFP fluorescence and 25 μg/ml hygromycin. Antibiotics of geneticin and hygromycin were bought from Sangon Biotech (Shanghai, China).

### Digital gene expression profiling analysis

Fungal samples of the wild-type strain and Δ*VdMcm1-5* used for digital gene expression profiling analysis were collected from 14-day-old cultures grown on BM, as described by Xiong et al. (2014). Total RNA was extracted using Trizol Reagent (Invitrogen, USA) and further purified with a PureLink RNA Mini Kit (Ambion, USA). After RNA quantification and qualification, two single-end sequencing libraries were prepared with NEBNext® Ultra™ RNA Library Prep Kit for Illumina® (NEB, USA) with average insert size of 150–200 bp. Sequencing was performed on an Illumina Hiseq2500 platform using 50 bp single-end reads at Beijing Novogene, Beijing, China (http://www.novogene.com/index.php).

After the removal of the adapter, ploy-N, and low-quality sequences, reads were aligned to the reference genome of *V. dahliae* VdLs.17 (Broad Institute) using TopHat (v2.0.9). HTSeq v0.6.1 was used to count the numbers of reads mapped to each gene. Then the RPKM of each gene was calculated based on the length of the gene and the numbers of reads mapped to the gene. Differentially expressed genes were selected using the DEGSeq R package (1.12.0) with the corrected *p* ≤ 0.005 (adjusted by the Benjamini & Hochberg method) and |Log_2_(fold-change)| > 1.5. The gene-function annotation was conducted based on Gene Ontology (GO) and Kyoto Encyclopedia of Genes and Genomes (KEGG) database. The significantly enriched KEGG pathways were selected using KOBAS (2.0) software with *q* < 0.05.

### Analysis of the secondary metabolism gene clusters

The secondary metabolism gene clusters in *V. dahliae* were predicted by the Secondary Metabolite Unknown Region Finder (SMURF; Khaldi et al., 2010). The orthologous genes in *Verticillium alfalfae* were identified with the BLASTP program by searching in the Verticillium genome database in Joint Genome Institute. The sequence data and annotated information were also acquired from Verticillium genome database (Klosterman et al., 2011). Synteny analysis of the two secondary metabolism gene cluster regions between *V. dahliae* and *V. alfalfae* was performed using GATA (Nix and Eisen, 2005). The RNA-Seq coverage data were visualized using Integrative Genomics Viewer (Robinson et al., 2011). The heatmap was drawn using MultiExperiment Viewer (Saeed et al., 2003).

### Quantitative real-time PCR assays

For quantitative real-time PCR (qRT-PCR) assays, cDNA was synthesized using oligo(DT)_18_ primer and SuperScript III Reverse Transcriptase (Invitrogen, USA). qRT-PCR reactions were performed using SuperReal PreMix Plus (Tiangen, China) with the Applied Biosystems 7500 Real-Time PCR system. The β-tubulin gene was used as an internal reference for all qRT-PCR analyses. Relative expression levels were calculated with 2^−ΔΔCt^ method (Livak and Schmittgen, 2001). All primers used in the present study are listed in Table S3.

### Pathogenicity assays

For pathogenicity tests, conidia were harvested from 7-day-old cultures grown in liquid CM by filtration through two layers of Miracloth and resuspended at 10^6^ conidia/ml in sterile distilled water. The roots of the 1-year-old smoke tree seedlings were inoculated with conidial suspensions for 10 min. Control plants were mock-inoculated with sterile distilled water. All inoculated smoke trees were then replanted into the soil. Ten smoke tree seedlings were tested per strain, and the experiment was repeated three times. For better observation, 1-month-old tobaccos were also inoculated with 10^6^ conidia/ml conidial suspensions for 1–3 days, and the GFP-expressing strains of wild-type and Δ*VdMcm1-5* used for tobacco infection tests were named as WT-GFP and Δ*VdMcm1-5-GFP*. For scanning electron microscopy, the freshly isolated roots of the smoke trees were inoculated with 10^7^ conidia/ml conidial suspensions for 24 h at room temperature, the water was sopped up with filter papers, then the dried roots were sputtered with Au and used for observation. To examine the ability of conidial adhesion, 10^5^ conidia of each strain were spread over the Hybond™-N^+^ membranes (GE Healthcare, UK), which were overlaid onto PDA plates at room temperature, and these membranes were then gently washed with 1 ml sterile distilled water after 18 h incubation. Approximately 750 μl conidial suspensions were obtained after gentle washing, and 50 μl conidial suspensions were used to coat the PDA plate. The number of colonies was determined after 5 days of growth on PDA at room temperature. The experiments were repeated three times.

## Results

### Identification of *VdMcm1*

**Figure 1:**
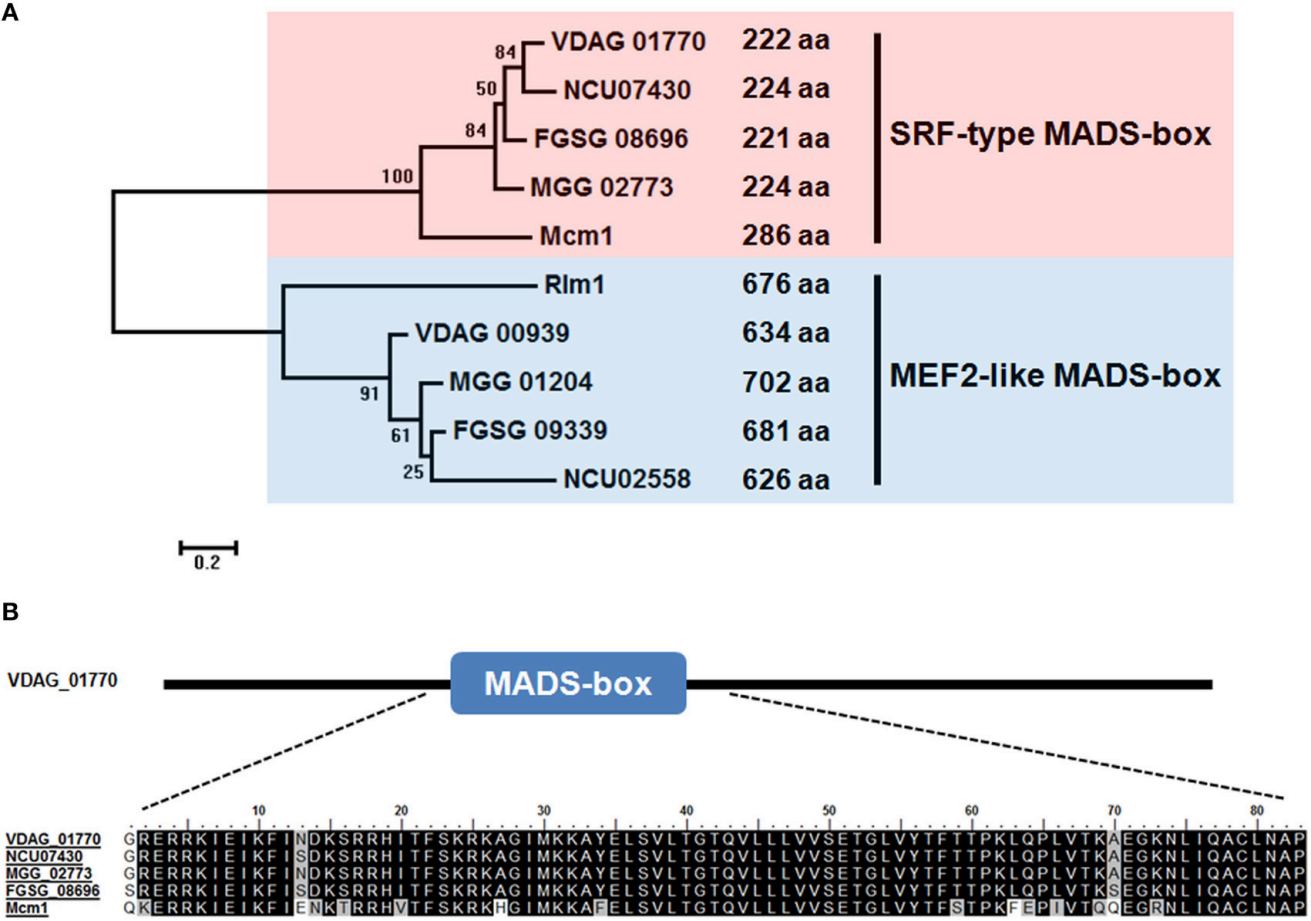
**Sequence analysis of ***VdMcm1***. (A)** Phylogenetic tree of MADS-box genes in *Verticillium dahliae* and its homologs from *Saccharomyces cerevisiae* (*Mcm1, Rlm1*), *Neurospora crassa* (NCU07430, NCU02558), *Magnaporthe oryzae* (MGG_02773, MGG_01204), *Fusarium graminearum* (FGSG_08696, FGSG_09339). VDAG_01770 and VDAG_00939 are homologs of *Mcm1* and *Rlm1* in *V. dahliae*, respectively. **(B)** Amino acid sequence alignment of the MADS-box domain of *VdMcm1* and its homologs from other fungal species.

We examined the *V. dahliae* genome database (JGI) and identified a gene (VDAG_01770) encoding 222 amino acids (aa) protein, which was homologous to *S. cerevisiae* Mcm1 (54.05% overall identity). Here, VDAG_01770 was designated as *VdMcm1*. *VdMcm1* also shared high degree of homology with *Mcm1* from *M. oryzae* (80.18%) and *F. graminearum* (78.08%). Phylogenetic analysis suggested that fungal MADS-box genes were divided into two subfamilies: SRF-type (average size about 235 aa) and MEF2-like (average size about 644 aa; Figure 1A). Herein, *VdMcm1* belongs to the SRF subfamily. Multiple sequence alignment confirmed that MADS-box domains of *Mcm1* orthologs from *V. dahliae, S. cerevisiae, M. oryzae, Neurospora crassa*, and *F. graminearum* are highly conserved (Figure 1B).

### *VdMcm1* contributes to hyphal growth but not to fungal biomass

**Figure 2:**
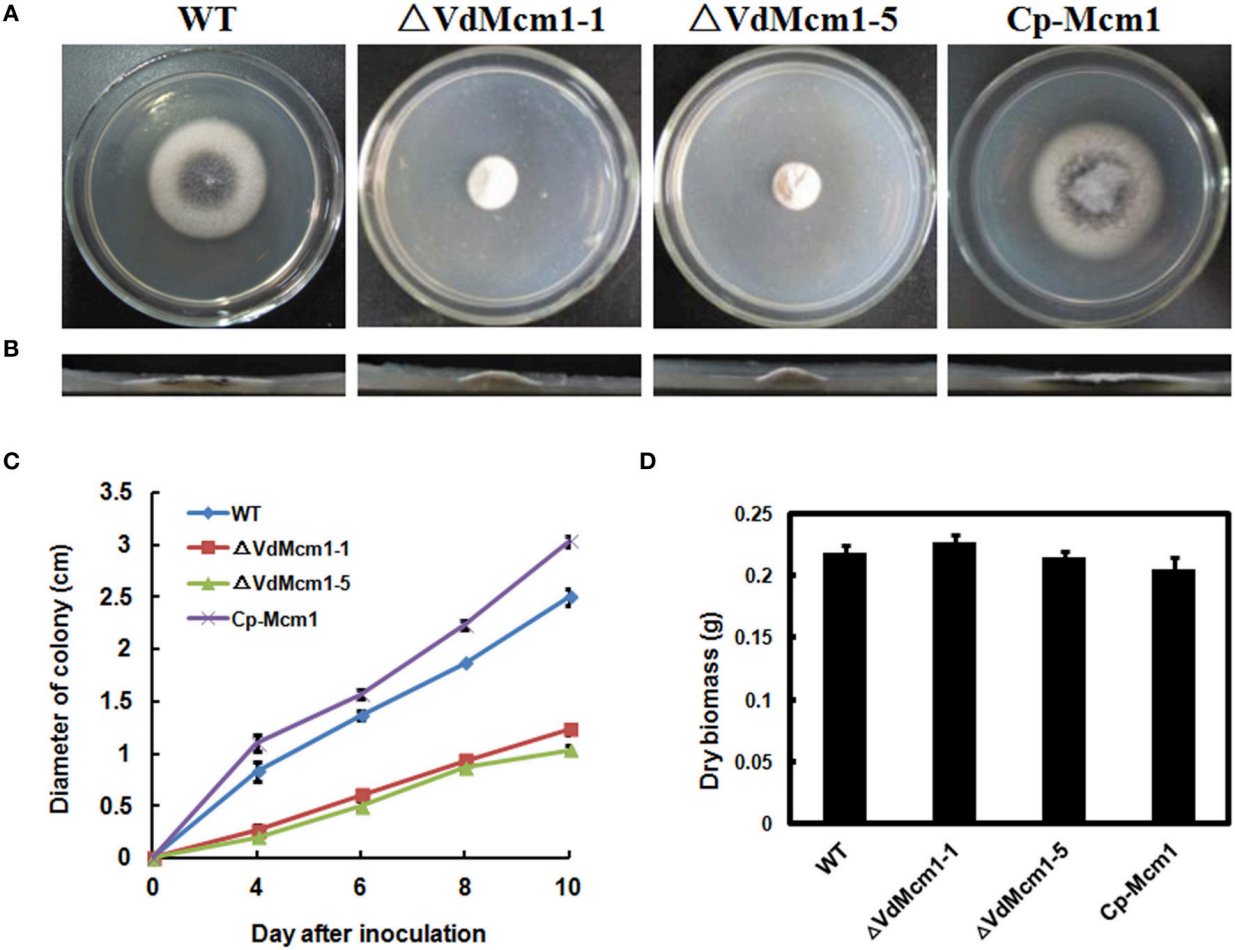
*****VdMcm1*** disruption results in reduced fungal growth. (A)** Colony morphology of wild-type strain, *VdMcm1* deletion mutants (Δ*VdMcm1-1*, Δ*VdMcm1-5*), and the complemented strain after 10 days of growth on PDA plates. **(B)** Vertical dissection of the colonies on PDA plates. **(C)** Radial growth rate of the strains on PDA plates. **(D)** Relative dry biomass of the indicated strains grown in liquid CM for 9 days at 25°C. The error bars represent standard deviations. The experiments were performed in triplicate.

In order to study the role of *VdMcm1* in *V. dahliae*, the entire open reading frame of the gene was replaced with a geneticin-resistant cassette, which generated three Δ*VdMcm1* deletion mutants (Δ*VdMcm1-1*, Δ*VdMcm1-5, and* Δ*VdMcm1-7*) with split-marker method (Figures S1A–D). Complementation strains were generated by introducing the *VdMcm1* gene with the putative native promoter region (2031 bp) and terminator region (1293 bp) into the Δ*VdMcm1-5* mutant, which had been confirmed by Southern blot (Figure S1E). Deletion mutant Δ*VdMcm1-5* and complemented strain (*Cp-Mcm1*) were selected for further analysis. Deletion mutants of *VdMcm1* showed significant reduction of hyphal growth on PDA plates (over 50% reduction; Figures 2A,C), and the aerial hyphae of the mutants were more compact than those of the wild-type and complemented strains (Figures 2A,B). However, biomass production of Δ*VdMcm1* mutants in liquid medium was similar to that of the wild-type and complemented strains (Figure 2D).

### *VdMcm1* deletion leads to reduced conidiation and aberrant conidial morphology

**Figure 3:**
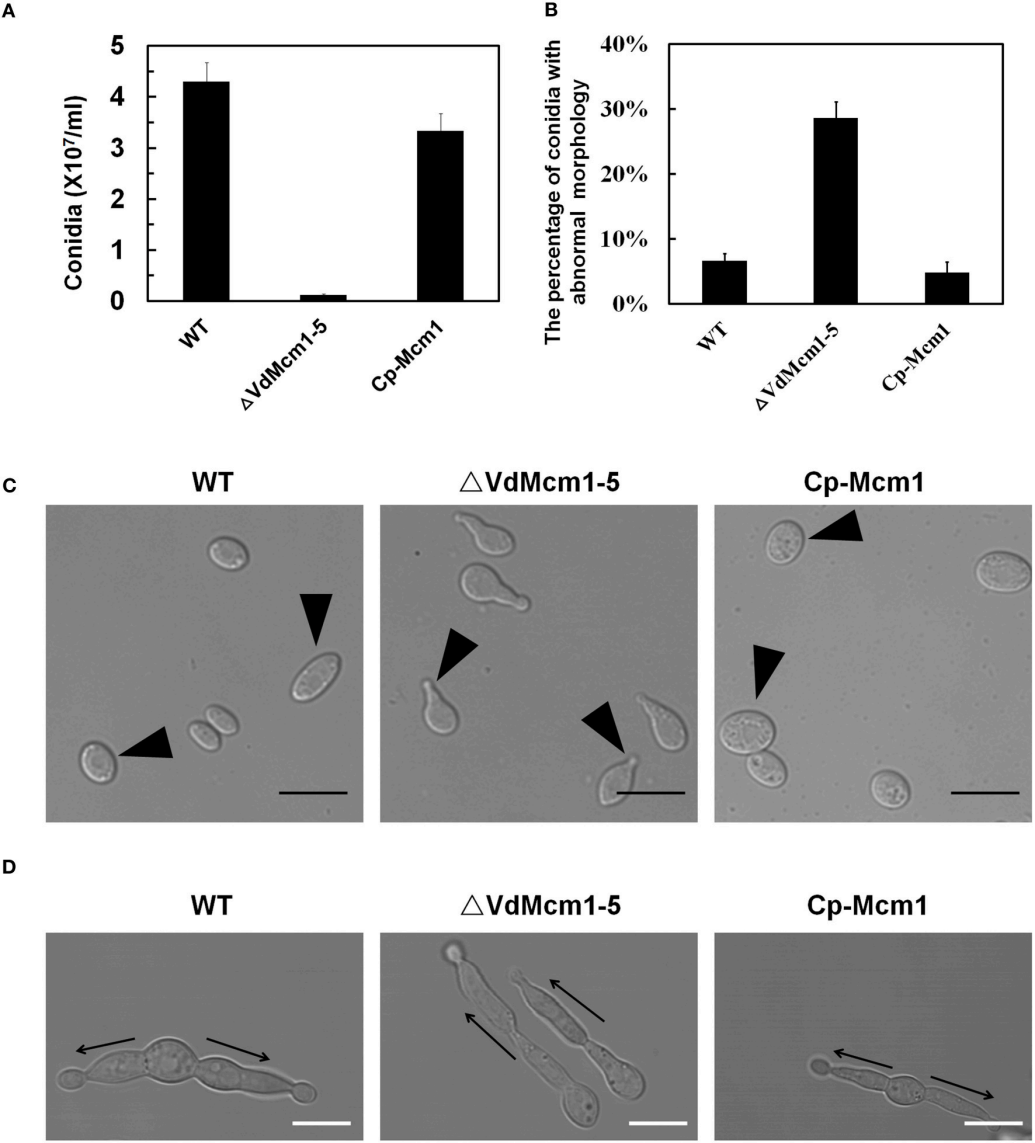
**Deletion of ***VdMcm1*** leads to reduced conidial production and abnormal conidial morphology. (A)** Conidial production of the wild-type strain, *VdMcm1* deletion mutant, and complemented strain after 10 days of growth on PDA plates. **(B)** The percentage of conidia with abnormal morphology in each strain. **(C)** Conidial morphology of wild-type strain, *VdMcm1* deletion mutant, and complemented strain. Conidia were harvested from cultures in liquid CM for 7 days. The arrowheads indicated normal conidia of wild-type and complemented strains, and abnormal conidia with the protuberance of *VdMcm1-5*. The scale bars represent 10 μm. **(D)** Germinated conidia of the wild-type strain, *VdMcm1* deletion mutant, and complemented strain after 24 h inoculated in YEPD medium. Arrows indicate growth direction. The scale bars represent 5 μm. The error bars represent standard deviations. The experiments were performed in triplicate.

Production of conidia in Δ*VdMcm1-5* was decreased by 80% compared with that in the wild-type and complemented strains (Figure 3A). Moreover, we found that, in addition to the traditional oval shape of conidia, about 30% conidia produced by the *VdMcm1* deletion mutant displayed a polar protrusion (Figures 3B,C). We also noticed that germ tube was only growing along the polar protrusion in the *VdMcm1* deletion mutant, whereas most (over 95%) conidia of wild-type and complemented strains exhibited a bidirectional germination (Figure 3D). These results suggest that *VdMcm1* is involved in the polar germination of conidia.

### *VdMcm1* is involved in cell wall integrity

**Figure 4:**
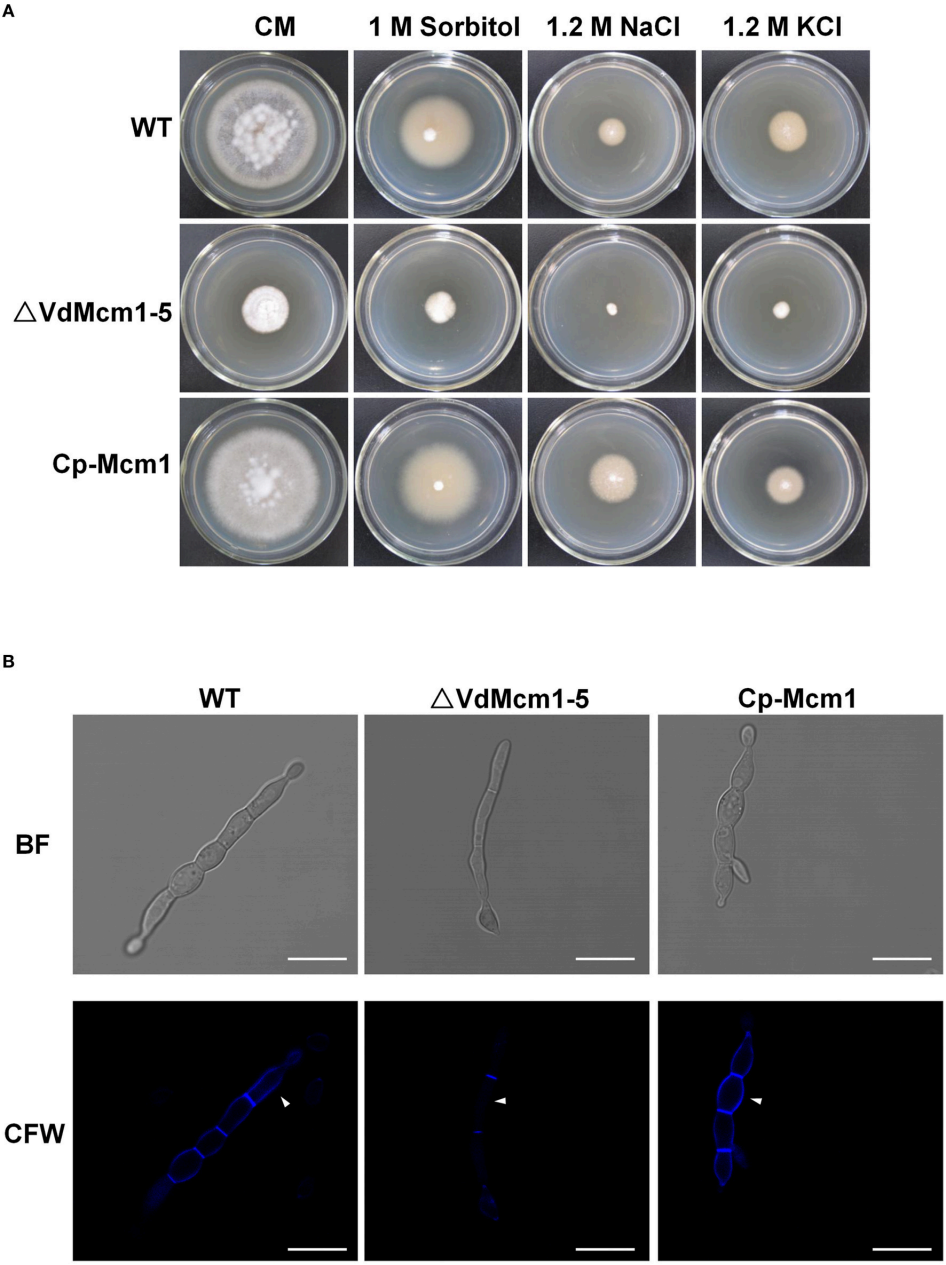
**The effect of ***VdMcm1*** on cell wall integrity. (A)** Colony morphology of the wild-type strain, Δ*VdMcm1-5* and complemented strain after 15 days of growth on CM or CM containing 1 M Sorbitol, 1.2 M NaCl, and 1.2 M KCl. **(B)** Germinated conidium stained with 10 μg/ml Calcofluor White. The white arrowheads indicate the cell wall stained with Calcofluor White. The scale bars represent 10 μm.

To test their sensitivity to osmotic stresses, all the strains were cultivated on CM containing 1 M sorbitol, 1.2 M NaCl, and 1.2 M KCl. The results in Figure 4A showed that the Δ*VdMcm1-5* displayed slightly increased sensitivity to osmotic stress, especially caused by NaCl and KCl. Relative growth was determined based on the colony diameter at 15 days post-inoculation. Following data analysis using *t*-test, the difference was considered significant at *p* < 0.05 (Table 1). To characterize whether VdMcm1 affected the properties of cell wall, CFW staining was used to monitor the chitin deposition in the fungal cell wall. It was obvious that the intensity of CFW staining of cell wall of the wild-type and complemented strains was much higher than that of the Δ*VdMcm1-5* strain, suggesting that chitin deposition is reduced in the cell wall of the Δ*VdMcm1-5* (Figure 4B).

**Table 1:** **The radial growth of wild type and Δ***VdMcm1-5*** mutant in response to osmotic stresses with 1 M Sorbitol, 1.2 M NaCl, 1.2 M KCl**.

**Compound**	**WT**		Δ***VdMcm1-5***	
	**Colony diameter (cm)**	**% growth**	**Colony diameter (cm)**	**% growth**
Untreated	4.20 ± 0.28	100	1.90 ± 0.1	100
1 M Sorbitol	2.87 ± 0.06	68.3a	1.25 ± 0.07	65.8a
1.2 M NaCl	1.00 ± 0.14	23.8a	0.33 ± 0.06	17.4b
1.2 M KCl	1.45 ± 0.07	34.5a	0.57 ± 0.06	30.0b

*The % growth was determined by using radial growth of treated samples divided the radial growth of untreated controls (100). Different letters “a, b” in the same row indicate significant differences at p < 0.05 and the same letters indicate no significant differences, which were calculated using t-test based on three repetitions*.

### *VdMcm1* affects microsclerotia formation and melanin biosynthesis

**Figure 5:**
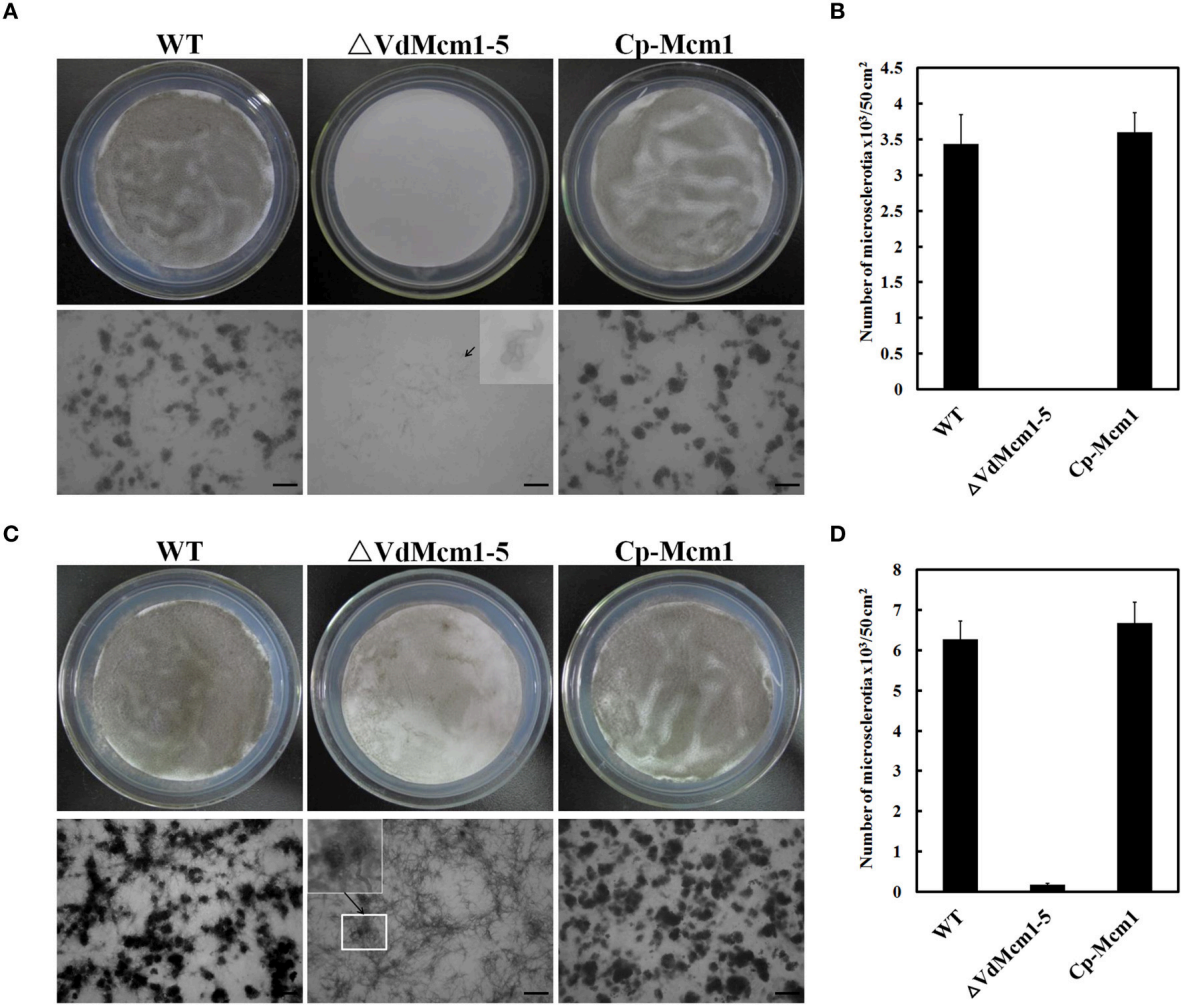
**Loss of ***VdMcm1*** causes defects in microsclerotia formation. (A)** Microsclerotia formation on BM plates with 10^5^ conidia spreading over the cellulose membrane after 6 days of growth. The enlarged view shows the unmelanized and swollen hyphae of Δ*VdMcm1-5*. The scale bars represent 100 μm. **(B)** The histogram represents the number of microsclerotia formed by the wild-type strain, Δ*VdMcm1-5*, and complemented strain on the cellulose membrane (φ = 80 mm) after 6 days of growth. **(C)** Microsclerotia formation on BM plates after 8 days of incubation. The enlarged view showed the microsclerotia of Δ*VdMcm1-5*. The scale bars represent 100 μm. **(D)** The histogram represented the number of microsclerotia formed by wild-type strain, Δ*VdMcm1-5*, and complemented strain on the cellulose membrane (φ = 80 mm) after 8 days of incubation. The error bars represent standard deviations. The experiments were performed in triplicate.

To examine whether the Δ*VdMcm1* deletion strain was defective in microsclerotia production, we observed the microsclerotia formation on BM. After 6 days post-inoculation (dpi), the Δ*VdMcm1-5* failed to produce melanized microsclerotia while the wild-type and complemented strains formed melanized microsclerotia (Figure 5A). Under microscopic examination at 6 dpi, about 3000/50 cm^2^ microsclerotia were formed in the wild-type and the complemented strains, whereas no microsclerotia were produced in Δ*VdMcm1-5* (Figure 5B). However, a small number of swollen, melanized hyphae were observed in the Δ*VdMcm1-5* (Figure 5B), and the melanin accumulation was significantly compromised in Δ*VdMcm1-5*. At 8 dpi, about 6000/50 cm^2^ highly melanized microsclerotia were formed in the wild-type and complemented strains (Figures 5C,D). In contrast, the Δ*VdMcm1-5* formed lots of swollen, melanized hyphae instead of microsclerotia, and the melanin accumulation was still significantly reduced in Δ*VdMcm1-5* compared with the wild-type and complemented strains (Figures 5C,D). In addition, the hyphae of Δ*VdMcm1-5* scarcely gathered together to form microsclerotia. At 14 dpi, the Δ*VdMcm1-5* still had significant defects in microsclerotia formation compared with the wild-type and complemented strains (data not shown). Consistent with reduced melanin accumulation in the Δ*VdMcm1-5* strain (Figures 2A, 5), genes related to melanin biosynthesis were downregulated in Δ*VdMcm1-5* (Figure S2). The results suggest that *VdMcm1* positively control microsclerotia formation and melanin biosynthesis.

### *VdMcm1* is involved in *s*urface hydrophobicity

**Figure 6:**
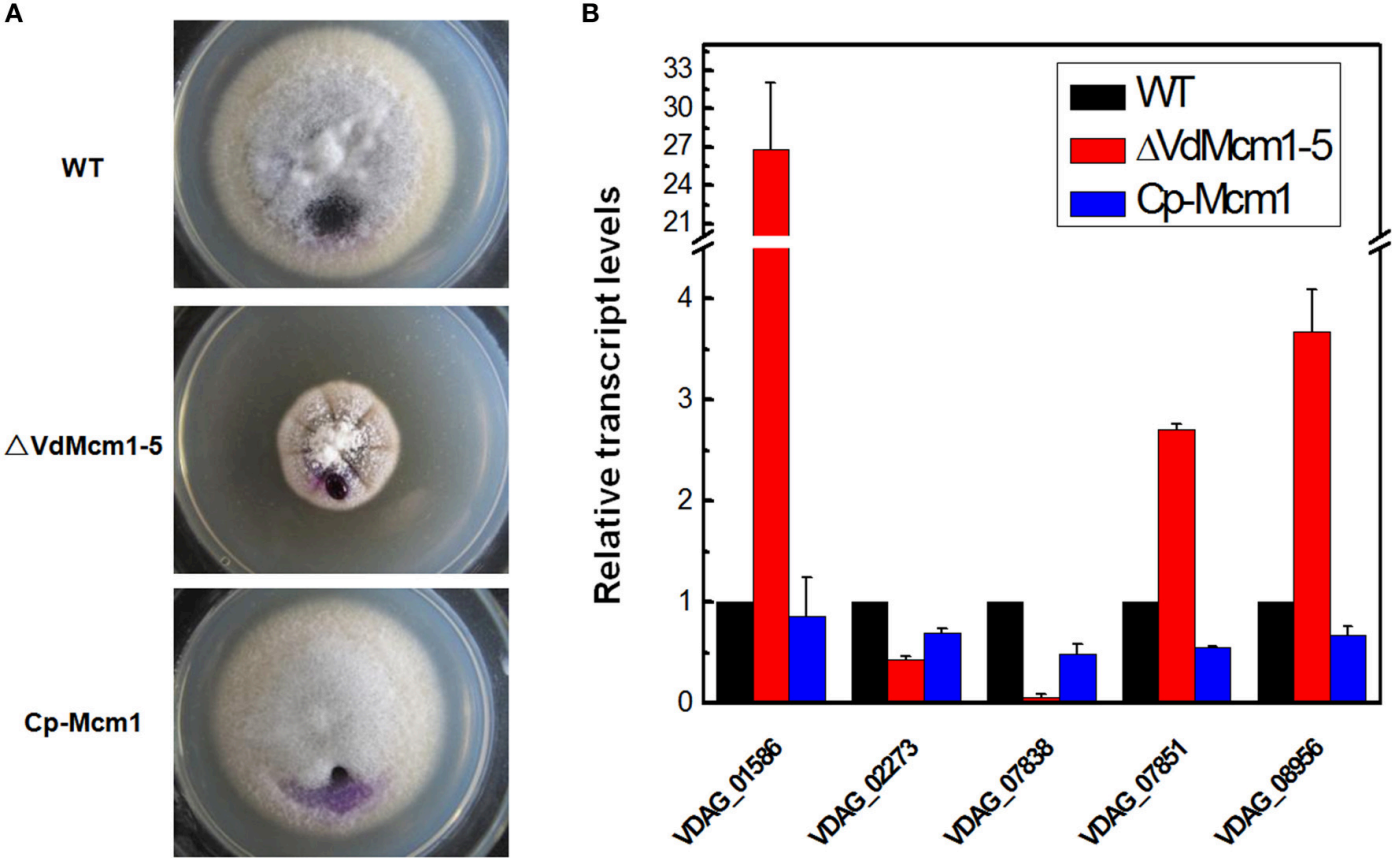
**Surface hydrophobicity assay and gene expression analysis. (A)** Surface hydrophobicity was tested by spotting 20 μl water droplets containing 0.12% bromophenol blue on the surface of 16-day-old colonies grown on PDA plates. The pictures were taken after 1 h of incubation at room temperature. **(B)** Relative gene expression levels of five hydrophobin genes by quantitative real-time PCR. Total RNA of the wild-type strain, Δ*VdMcm1-5*, and complemented strain was extracted from the 16-day-old cultures grown on PDA. The β-tubulin gene was used as an internal reference. The error bars represent standard deviations. The experiments were performed in triplicate.

As mentioned above, aerial hyphae in Δ*VdMcm1* mutants were more compact than those of the wild-type and complemented strains. To examine whether the surface hydrophobicity was affected after *VdMcm1* deletion, we tested the surface hydrophobicity by placing a drop on the surface of colonies. The results showed that the drop remained suspended on the colony surface of the Δ*VdMcm1-5*, whereas it was soaked into the surface of the wild-type and complemented strains after 1 h (Figure 6A), indicating that *VdMcm1* is a negative regulator of surface hydrophobicity.

To elucidate the molecular mechanism underlying the responses to surface hydrophobicity, five genes (VDAG_01586, VDAG_02273, VDAG_07838, VDAG_07851, and VDAG_08956) encoding hydrophobins in the *V. dahliae* genome were selected for expression analysis. Total RNA was extracted from the 16-day-old cultures grown on PDA. Transcript levels of three genes (VDAG_01586, VDAG_07851, and VDAG_08956) were highly increased in the Δ*VdMcm1-5* compared with the wild-type and complemented strains, especially VDAG_01586 (fold-change > 25) and VDAG_08956 (fold-change > 3; Figure 6B). It indicated that these genes might play fundamental roles in surface hydrophobicity. In contrast, VDAG_02273 (over 50% reduction) and VDAG_07838 (over 90% reduction) were downregulated in the Δ*VdMcm1-5* (Figure 6B). The results suggest that *VdMcm1* is involved in surface hydrophobicity by regulating the expression of hydrophobin encoding genes.

### *VdMcm1* deletion mutant shows attenuated virulence

**Figure 7:**
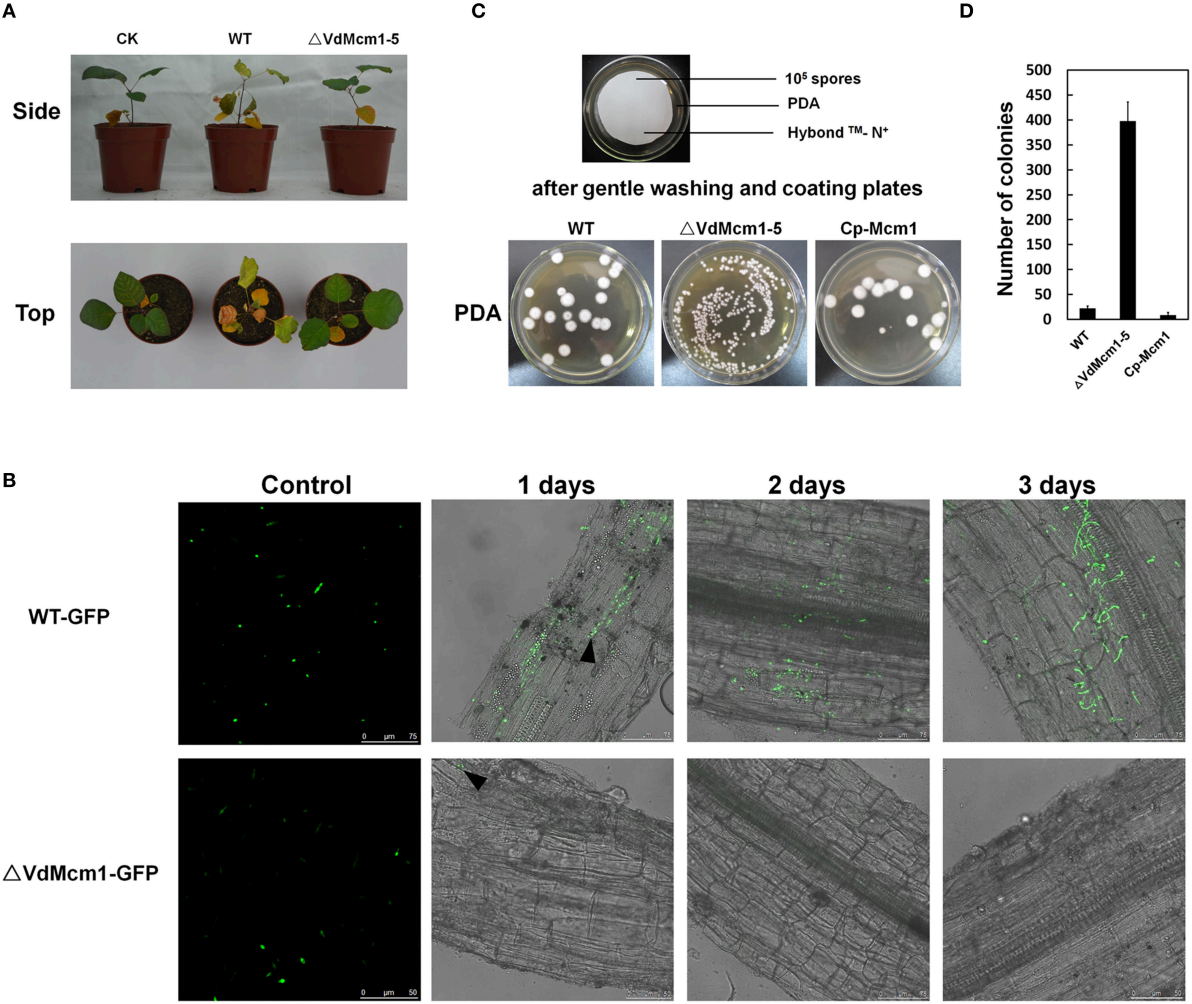
**Pathogenicity assays of ***VdMcm1*** deletion mutants on smoke trees and tobacco seedlings. (A)** Side and top views of one-year-old smoke tree seedlings inoculated with 10^6^ spores/ml conidial suspension of the wild-type and Δ*VdMcm1-5* for 10 min. The smoke tree seedlings in CK were inoculated with distilled water for 10 min. The pictures were taken at 30 days after inoculation. **(B)** Microscope observation of 1-month-old tobacco roots inoculated with GFP-expressing wild-type strain (WT-GFP) and Δ*VdMcm1-5* (Δ*VdMcm1-GFP*), respectively. 10^6^ conidia/ml of WT-GFP, Δ*VdMcm1-GFP* were used. The arrowheads indicated adherent conidia on the root epidermis. The scale bars represent 75 μm. **(C)** Conidial adhesion tests. 10^5^ conidia harvested from liquid CM were grown on Hybond™-N^+^ membranes that were overlaid on PDA plates for 18 h. 1 ml distilled water was used to gently wash through the membranes and repeated five times, and about 750 μl conidial suspensions were acquired. Fifty microliter conidial suspensions were spread over the PDA plates. The pictures were taken after 5 days of growth. **(D)** The number of colonies grown on PDA plates after 5 days. The error bars represent standard deviations. The experiments were performed in triplicate.

The pathogenicity assays showed that smoke tree seedlings inoculated by the Δ*VdMcm1-5* exhibited slight chlorosis at 30 dpi (Figure 7A). In contrast, the smoke tree seedlings inoculated with the wild-type strain showed obvious wilt symptoms (Figure 7A). Furthermore, we examined the conidial adhesion on the root of the smoke tree at 1 dpi with scanning electron microscopy. Conidia of the wild-type and complemented strains were attached to root epidermis and germinated, whereas it was hard to see the conidia or germinated conidia on the root epidermis inoculated with Δ*VdMcm1-5* (Figure S3). This suggests that *VdMcm1* may be involved in the adhesion process during plant infection.

To observe the infection process in details, GFP-expressing wild-type (WT-GFP) and Δ*VdMcm1-5* (Δ*VdMcm1*-GFP) strains were used. Conidia of the WT-GFP strain could attach, germinate, and spread onto the root while conidia of Δ*VdMcm1*-GFP strain almost failed to adhere onto the root (Figure 7B). In addition, the adhesion ability was tested by gently washing the conidia inoculated on the membrane, which was overlaid on the PDA plates after 18 h. The results showed that conidia of the Δ*VdMcm1-5* were more likely to be washed away from the membrane than those of the wild-type and complemented strains. After inoculation on PDA plates, 398 colonies were recovered from the Δ*VdMcm1-5* while 22 and 9 colonies were recovered from the wild-type and complemented strains, respectively (Figures 7C,D). Taking all the results shown above into consideration, we hypothesize that that VdMcm1 is necessary for the conidial attachment during the infection process.

### Comparative transcriptomic analysis of Δ*VdMcm1* and wild type

**Figure 8:**
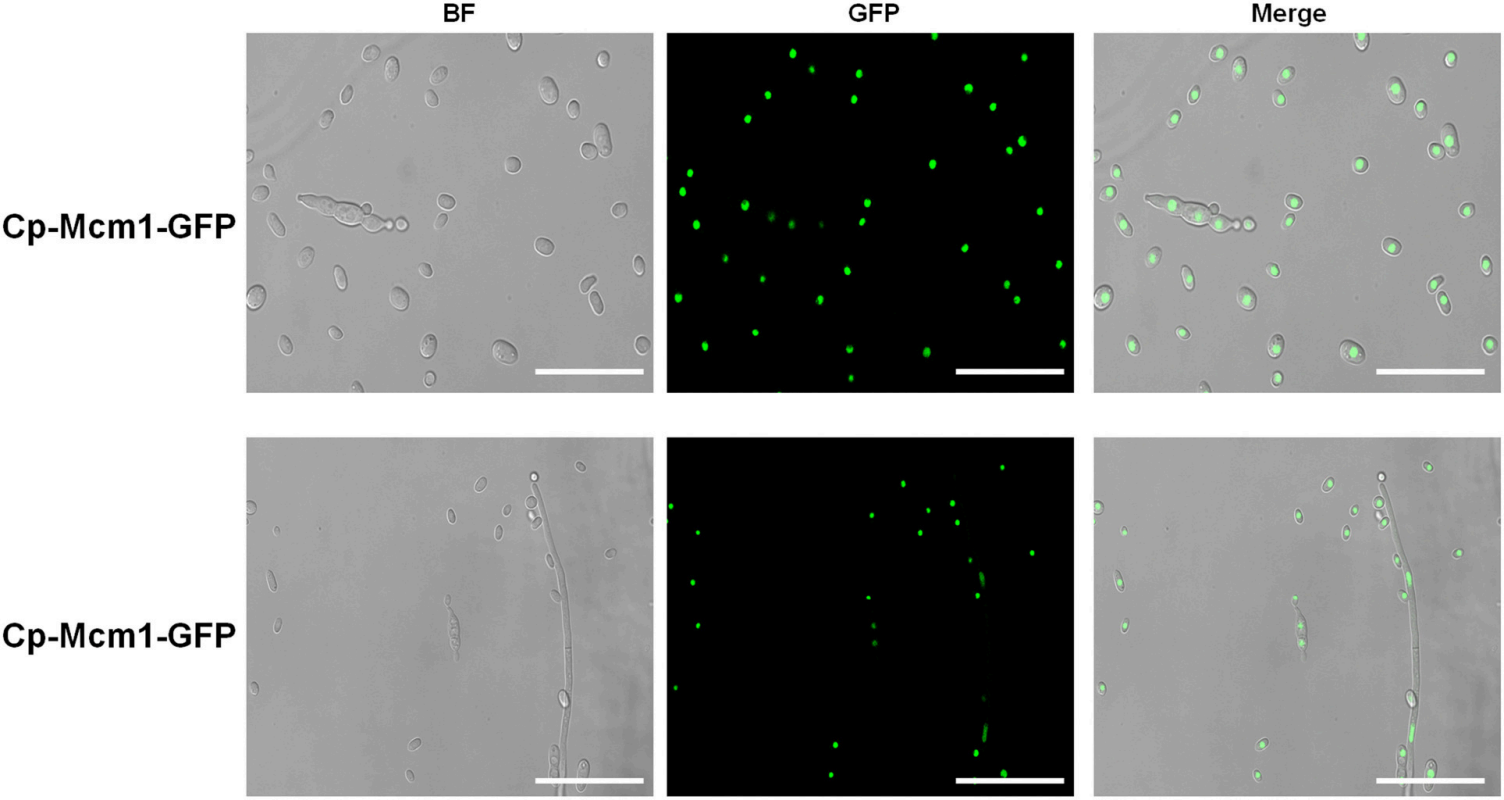
**Subcellular localization of VdMcm1**. Confocal microscopy of *Cp-Mcm1-GFP* (*VdMcm1-GFP* fusion, containing native promoter and coding sequence of VdMcm1 without stop codon and GFP fragment, was introduced into the *VdMcm1* deletion mutant) conidia and hyphae, which were harvested from liquid CM. The fluorescence was mainly localized in the nucleus. The scale bars represent 25 μm.

**Figure 9:**
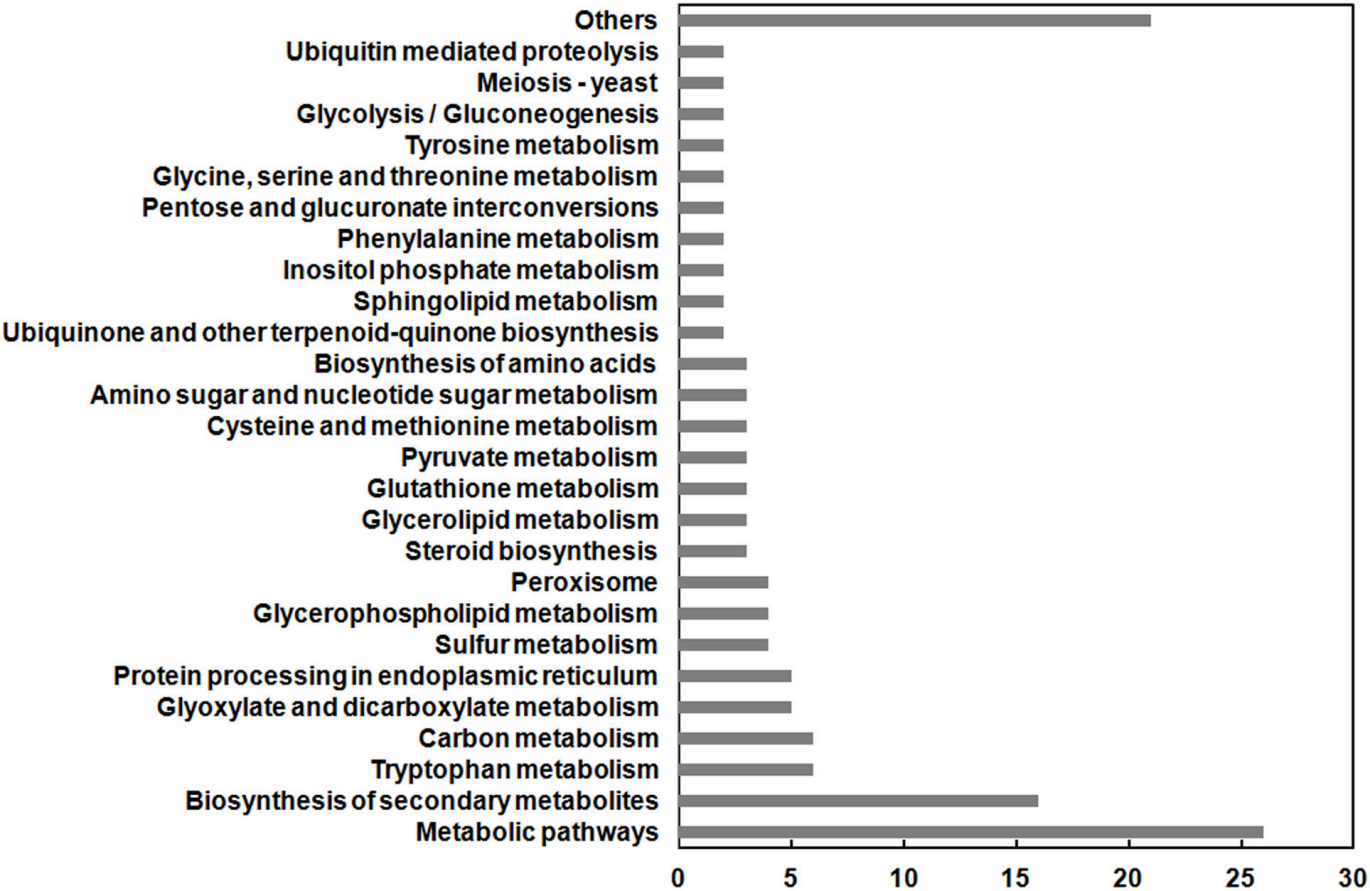
**Functional categories of gene significantly downregulated in Δ***VdMcm1-5*****. The histogram shows the enriched KEGG pathways. The values of *x*-axis represent the number of genes in each category.

As a conserved transcription factor, Mcm1 is expected to be localized in the nucleus to regulate gene expression. Consistently, VdMcm1 was observed in the nucleus (Figure 8). To identify genes potentially regulated by *VdMcm1*, the comparative transcriptomic analysis was conducted between the wild-type strain and Δ*VdMcm1-5*. Overall, differential expression analysis showed that 351 genes were upregulated and 823 genes were downregulated in Δ*VdMcm1-5* compared with the wild-type strain. Functional analysis based on GO and KEGG pathways annotation revealed that the 823 downregulated genes were involved in various cellular processes, such as biosynthesis of secondary metabolism, amino acid metabolism, and pyruvate metabolism (Figure 9). Transcriptomic data were confirmed by qRT-PCR to determine the expression levels of several genes (two bZIP genes VDAG_08640 and VDAG_08676, a Crz1 homology gene VDAG_03208, a melanin biosynthesis related gene VDAG_00190, a pyruvate kinase gene VDAG_01206). The results showed that qRT-PCR results and transcriptomic data were well correlated (Figure S4).

As mentioned above, the Δ*VdMcm1-5* mutant showed less chitin deposition of cell wall than that of the wild-type and complemented strains. Consistent with the phenotype, several important genes involved in chitin biosynthesis had significantly reduced expression levels in Δ*VdMcm1-5* mutant, such as VDAG_08591 (chitin synthase), VDAG_02580 (chitin synthase), VDAG_03141 (chitin synthase), VDAG_00376 (chitin synthase D).

**Figure 10:**
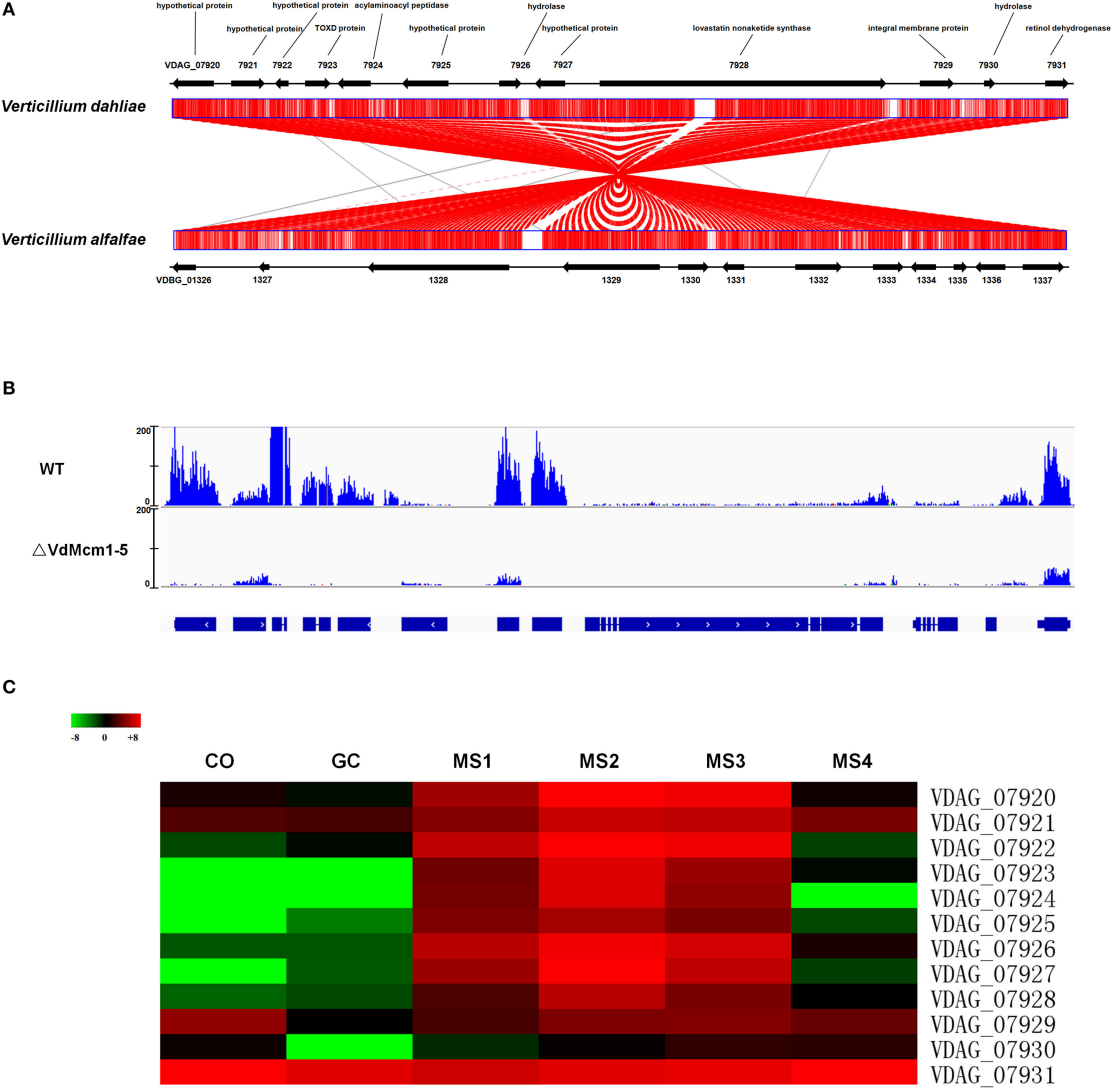
*****VdMcm1*** regulates genes expression of a secondary metabolism gene cluster. (A)** Structure and gene annotation of the secondary metabolism gene cluster in *V. dahliae and V. alfalfae*. This gene cluster of *V. dahliae* showed high homology to that of *V. alfalfae*. Synteny was indicated by red lines. **(B)** Visualization of RNA-Seq coverage of this secondary metabolism gene cluster in wild-type strain and *VdMcm1* deletion mutant. The blue curves indicated reads coverage. **(C)** Heatmap showing gene expression profiles of genes in the cluster during microsclerotia development. CO and GC represent conidia and conidia germination; MS1–MS4 represents four typical stages during the entire process of microsclerotia formation at 60, 72, 96 h, and 14 days. Log_2_(FPKM) value was used to draw the picture. Green color represented the negative value of Log_2_(FPKM), and red color represented the positive value of Log_2_(FPKM). Black color represented the zero value of Log_2_(FPKM).

Combining RNA-Seq data with genome-wide analysis of genes involved in fungal secondary metabolites, we found that six core backbone genes related to secondary metabolism process were regulated by VdMcm1. Among the six genes, five genes (VDAG_03466, VDAG_07928, VDAG_09624, and VDAG_09802) were significantly downregulated in the Δ*VdMcm1-5* while one gene (VDAG_03964, surfactin synthetase) was significantly upregulated (Table 2). Further, analysis showed that the entire gene cluster containing VDAG_07928 and other 11 genes (Figure 10A) was significantly downregulated in Δ*VdMcm1-5* compared with the wild-type strain (Figure 10B). Additionally, VDAG_07923, VDAG_07924, VDAG_07926, VDAG_07928, VDAG_07929, VDAG_07930, and VDAG_07931 in the cluster encoded TOXD protein, acylaminoacyl peptidase, hydrolase, lovastatin nonaketide synthase, integral membrane protein, hydrolase, and retinol dehydrogenase, respectively (Figure 10A). Comparative analysis also showed that this cluster exhibited high synteny between *V. dahliae* and *V. alfalfae*. However, this cluster was not found in other plant pathogenic fungi, such as *F. graminearum* and *M. oryzae*. Interestingly, VDAG_07928 was identified to be the only hybrid PKS and NRPS protein in the genome of *V. dahliae*. Nine out of 12 genes in this cluster, from VDAG_07920 to VDAG_07928, were significantly upregulated during microsclerotia development (Figure 10C). Two additional secondary metabolism gene clusters also showed similar expression pattern of downregulation in *VdMcm1* deletion mutant and during microsclerotia formation (Figure S5).

**Table 2:** **Putative secondary metabolism gene clusters and their expression in ***V. dahlia*****.

**Backbone gene_id**	**Annotated_gene_function**	**Cluster**	**SMURF prediction**	**VdMcm1 vs. WT [Log2(fold change)]**
**VDAG_00190.1**	Conidial yellow pigment biosynthesis polyketide synthase	NA	PKS	–3.1161
VDAG_01835.1	Fatty acid synthase S-acetyltransferase	From VDAG_01835 to VDAG_01842	PKS	NA
**VDAG_01856.1**	Phenolpthiocerol synthesis polyketide synthase ppsA	NA	PKS	NA
VDAG_02144.1	L-aminoadipate-semialdehyde dehydrogenase	From VDAG_02132 to VDAG_02147	NRPS-Like	NA
VDAG_03466.1	Fatty acid synthase S-acetyltransferase	From VDAG_03465 to VDAG_03470	PKS	–3.2949
VDAG_03964.1	Surfactin synthetase subunit 3	From VDAG_03961 to VDAG_03970	NRPS	1.706
VDAG_05314.1	N-(5-amino-5-carboxypentanoyl)-L-cysteinyl-D-valine synthase	From VDAG_05314 to VDAG_05325	NRPS	NA
VDAG_06409.1	3-oxoacyl-[acyl-carrier-protein] synthase	From VDAG_06409 to VDAG_06415	PKS-Like	NA
VDAG_07270.1	Mycocerosic acid synthase	From VDAG_07259 to VDAG_07280	PKS	NA
VDAG_07928.1	Lovastatin nonaketide synthase	From VDAG_07920 to VDAG_07931	HYBRID	–3.4231
VDAG_08188.1	D-alanine-poly(phosphoribitol) ligase subunit 1	From VDAG_08174 to VDAG_08191	NRPS-Like	NA
VDAG_08448.1	Lovastatin nonaketide synthase	From VDAG_08443 to VDAG_08448	PKS	NA
VDAG_09534.1	Aflatoxin biosynthesis polyketide synthase	From VDAG_09526 to VDAG_09535	PKS	NA
VDAG_09624.1	Peroxisomal-coenzyme A synthetase	From VDAG_09620 to VDAG_09630	NRPS-Like	–1.9196
VDAG_09654.1	Hypothetical protein	From VDAG_09647 to VDAG_09663	NRPS-Like	NA
**VDAG_09763.1**	Enterobactin synthetase component F	NA	NRPS-Like	NA
VDAG_09802.1	Transferase family protein	From VDAG_09801 to VDAG_09802	NRPS-Like	–5.841
VDAG_10211.1	Hypothetical protein	From VDAG_10211 to VDAG_10219	NRPS-Like	NA

*Bold Gene ids meant that the corresponding cluster contain only one gene. The Log2(fold change) value, the significantly expressed genes were shown. SMURF, a software, was used to predict the secondary metabolism genes and gene clusters in genomic sequence data. NA represented that genes were not differentially expressed in VdMcm1 deletion mutant compared with wild-type strain*.

Previously, a *V. dahliae* homolog of *M. oryzae* and *F. oxysporum* C_2_H_2_ transcription factor *Con7, Vta2* (VDAG_05685) was reported to be involved in fungal adhesion and virulence (Tran et al., 2014). In this study, we found that *Vta2* was significantly downregulated in Δ*VdMcm1-5*. Further, investigation revealed that 61 genes regulated by *Vta2* were significantly downregulated in the Δ*VdMcm1-5* as well (Table S1). The overlap of genes regulated by both *VdMcm1* and *Vta2* might be responsible for partially similar phenotypes of the Δ*VdMcm1* mutant and the Δ*Vta2* mutant, such as slow growth rate, reduced virulence and adhesion. However, as far as microsclerotia formation was concerned, *VdMcm1* and *Vta2* had an opposite effect. Among them, four genes important for early infection were found including pth11-like protein [VDAG_05358, a G protein-coupled receptor (GPCR) genes], and other three secreted proteins: secreted catalase-peroxidase (VDAG_04826), secreted adhesion genes (VDAG_07185), and one hypothetical protein (VDAG_01806). Further, investigation revealed that 102 secreted proteins with various functions, such as peptidases, glycoside hydrolases, and ligninases (Table S2), were significantly downregulated in Δ*VdMcm1-5* compared with the wild-type strain. Importantly, 30 genes encoded small (≤300 aa) and cysteine-rich (≥4 cysteine residues) proteins, which might be candidates for fungal effectors (Table S2). In addition, another 15 GPCR genes, which might be important for signal perception and transduction, were detected to be significantly downregulated in the Δ*VdMcm1-5* mutant.

Overall, VdMcm1 functioned as the main regulator of secondary metabolism, infection, and microsclerotia formation.

## Discussion

The MADS-box transcription factors are a conserved gene family that contains various numbers of representatives across eukaryotes. In general, there are two MADS-box transcription factors homologous to Mcm1 and Rlm1 of *S. cerevisiae* in filamentous fungi. Interestingly, *Mcm1* was essential in *S. cerevisiae*. However, Mcm1 orthologs were not essential in other filamentous fungi, such as *M. oryzae* (Zhou et al., 2011), *S. macrospora* (Nolting and Pöggeler, 2006), *Schizosaccharomyces pombe* (Didmon et al., 2002), *F. graminearum* (Yang et al., 2015), and *F. verticillioides* (Ortiz and Shim, 2013). In this study, the MADS-box transcription factor gene *VdMcm1* (VDAG_01770) was identified and characterized in *V. dahliae*. The results indicated that *VdMcm1* possesses pleiotropic functions and regulates fungal growth, microsclerotia formation, and virulence of *V. dahliae*.

Previous studies revealed that fungal cell morphology was closely related to chitin and glucan contents in fungal cell wall. In this study, we found that the conidia exhibited abnormal morphology in Δ*VdMcm1* mutants. In addition, the chitin deposition of cell wall was reduced and genes encoding chitin synthase were differentially downregulated in the Δ*VdMcm1* mutant, i.e., VDAG_08591 (FC > 2.5), VDAG_02580 (FC > 1.5), VDAG_03141 (FC > 1.5), VDAG_00376 (FC > 2.5). Additionally, VDAG_00511 (glucan 1,3-beta-glucosidase, FC > 5), VDAG_02814 (glucan 1,3-beta-glucosidase, FC > 5), and VDAG_05658 (chitinase, FC > 5) were also significantly reduced in the Δ*VdMcm1* mutant. The reduced expression levels of genes involved in cell wall integrity were possibly responsible for the abnormal morphology. In *M. oryzae*, deletion of *CHS1* led to severe defects in conidia morphology (over 90% conidia were abnormal; Kong et al., 2012). The C_2_H_2_ transcription factor Con7p was involved in conidial morphology in *M. oryzae* (Odenbach et al., 2007) and *F. oxysporum* (Ruiz-Roldán et al., 2015). Consistent with their morphological defects, chitin content was altered in Con7 deletion mutants in *M. oryzae* (Odenbach et al., 2007), and chitinase activity was drastically reduced in Con7 deletion mutant in *F. oxysporum* (Ruiz-Roldán et al., 2015). Interestingly, the conidia of Δ*VdMcm1* mutant showed similar morphological defects to the Con7 deletion mutants in *M. oryzae* and *F. oxysporum*. In addition, Con7 deletion mutants in *V. dahliae and F. oxysporum* were reduced in the conidial production compared with the wild-type strain (Tran et al., 2014), and the Δ*VdMcm1* mutant also showed defective conidial production.

For phytopathogenic fungi, conidial attachment, and germination are usually the initial steps of infection, and the signal perception or transduction is important for these processes. In this study, we found that conidia of Δ*VdMcm1-5* were hardly attached to the root, and a pth11-like GPCR protein (VDAG_05358, FC > 4.5) was significantly downregulated in the *VdMcm1* deletion mutant. The pth11-like protein was postulated to perceive the host surface signal during appressorium formation and was essential for pathogenicity in *M. oryzae* (DeZwaan et al., 1999). Furthermore, other 15 putative GPCR genes were also differentially downregulated in the *VdMcm1* deletion mutant, i.e., VDAG_02933 (Microbial Opsin), VDAG_00541 (Family C-like GPCR), and VDAG_03447 (Hlyll_domain GPCR). GPCRs convey the external signals to heterotrimeric G proteins, which then activate the cyclic adenosine monophosphate and mitogen-activated protein kinase pathways. These signaling pathways play roles in fungal growth, mating, and virulence (Servin et al., 2012).

Secreted proteins of plant pathogens play important roles in host infection and colonization, and some of them, known as effectors, showed critical functions during infection process (Oliva et al., 2010; de Jonge et al., 2011; Vargas et al., 2015). In the genome of *V. dahliae*, more than 700 secreted proteins were identified (Klosterman et al., 2011). In this study, significantly reduced expression levels of 102 putative secreted protein encoding genes were detected in Δ*VdMcm1-5* mutant compared with the wild-type strain. Some of them, such as VDAG_09343 (peptidases), VDAG_02733 (peptidases), VDAG_02906 (glycoside hydrolases) and VDAG_06155 (pectate lyase), might be important for the infection process as they might degrade the plant cell wall and could also act as fungal effectors due to small (≤300 aa) and cysteine-rich (≥4 cysteine residues) properties (Table S2).

Hundreds of putative target genes of *VdMcm1* were identified by comparative transcriptomic analysis between the wild-type strain and Δ*VdMcm1-5* mutant. Remarkably, dozens of downstream genes regulated by VdMcm1 were also identified as putative target genes of *Vta2* (Tran et al., 2014). Some phenotypes of the *VdMcm1* deletion mutant were similar to the *Vta2* deletion mutant. First of all, the growth rate of these two mutants was significantly reduced. Secondly, both mutants displayed drastic reduction of conidiation. Thirdly, the virulence of both mutants was reduced. However, the mechanism for the reduced virulence was different between these two mutants. Conidia of *VdMcm1* deletion mutants failed to attach onto the roots while the *Vta2* deletion mutants could adhere and germinate on the roots but not colonize host stems (Tran et al., 2014). Although the morphology of conidia was not mentioned in *Vta2* deletion mutant, the conidial morphology of *VdMcm1* deletion mutant was abnormal with a protuberance. Besides conidial morphology, the roles of *VdMcm1* and *Vta2* in microsclerotia formation are opposite. *VdMcm1* is a positive regulator in microsclerotia formation, whereas *Vta2* is a negative regulator. We believe that it will provide important insights into microsclerotia formation to study the relationship between VdMcm1 and Vta2, although it will be a big challenge.

Secondary metabolites play important roles in virulence in fungi. Fungal secondary metabolites are mainly polyketides, non-ribosomal peptides, terpenes, and indole alkaloids, and the genes responsible for their biosynthesis are usually arranged in clusters (Keller et al., 2005; Fox and Howlett, 2008). Among them, melanin (a polyketides) is one of the most noticeable secondary metabolites produced by *V. dahliae* that would continuously accumulate during microsclerotia development (Bell et al., 1976; Stipanovic and Bell, 1976; Duressa et al., 2013; Xiong et al., 2014). Here, the *VdMcm1* deletion mutants showed obvious defect in melanin accumulation, suggesting that *VdMcm1* is essential for melanin production. In addition, using comparative transcriptomics, we discovered that several putative secondary metabolism gene clusters, including the only one PKS/NRPS hybrid gene cluster in *V. dahliae*, were regulated by *VdMcm1*. More importantly, putative Mcm1 binding site (CC[T/C][A/T]_3_NN[A/G]G) was found in the promoter sequences of two PKS/NRPS hybrid cluster genes VDAG_07924 and VDAG_07927, which were not expressed in the *VdMcm1* deletion mutant (data not shown). *VdMcm1* orthologs in other fungi are also involved in secondary metabolism. For example, *FgMcm1* is involved in deoxynivalenol (DON) production, i.e., the deletion of *FgMcm1* leads to a significant reduction of DON production (Yang et al., 2015). The expression levels of gene clusters involved in secondary metabolites production were downregulated in the *FgMcm1* deletion mutant including PKS2, PKS5, PKS9, NRPS7, and NRPS 14 gene clusters (Yang et al., 2015). Similarly, *FvMcm1* deletion mutant was dramatically reduced (about 50%) in the Fumonisin B1 production and the expression of PKS related genes compared with the wild-type strain (Ortiz and Shim, 2013).

Microsclerotia play important roles in disease cycles. They germinate to hyphae and infect the host when conditions are suitable. In this study, we found that microsclerotia formation was dramatically reduced in *VdMcm1* deletion mutant. Consistent with the reduced microsclerotia formation, genes involved in this process were downregulated in *VdMcm1* deletion mutant, such as melanin biosynthesis genes VDAG_00189 and VDAG_03665, a fad binding domain-containing protein VDAG_01149, a cytochrome p450 VDAG_03650, and a bZIP transcription factor VDAG_08640 (Duressa et al., 2013; Xiong et al., 2014). Furthermore, a hydrophobin-encoding gene VDAG_02273 reported to be involved in microsclerotia formation (Klimes and Dobinson, 2006; Klimes et al., 2008) showed reduced transcript level in Δ*VdMcm1-5*. The results indicated that *VdMcm1* controls microsclerotia formation by regulating the downstream genes that are involved in microsclerotia development.

In summary, the results displayed in this study demonstrate that *VdMcm1* plays important roles in fungal development, secondary metabolism, and virulence in plant pathogenic fungus *V. dahliae*. The results provide evidence that *VdMcm1* plays pleiotropic roles through regulating hundreds of downstream genes. As for disease control, *VdMcm1* is a potential target to control the fungal growth, microsclerotia development, and virulence in *V. dahliae*, although the genetic and signaling networks related to the microsclerotia development and pathogenicity of *V. dahliae* remain unclear. The data presented in this study can facilitate the exploration of upstream activators and downstream effectors of VdMcm1 and explain how they function in microsclerotia formation and virulence.

## Author contributions

YW, CT, and DX designed the experiments. DX and LT performed the experiments and the data analyses. DX and YW prepared the figures and wrote the manuscript.

### Conflict of interest statement

The authors declare that the research was conducted in the absence of any commercial or financial relationships that could be construed as a potential conflict of interest.

## Availability of supporting data

DGE data were submitted to the NCBI SRA database (http://www.ncbi.nlm.nih.gov/Traces/sra/) with the accession number: SRR2087157 (wild type), SRR2087158 (Δ*VdMcm1-5*).

## Supplementary material

Figure S1 **The strategy of gene replacement and validation of gene deletion mutants. (A)** Gene replacement strategy for the deletion of *VdMcm1*. The split-marker method was used. The entire coding sequence of *VdMcm1* was replaced with a geneticin resistance cassette. **(B)** Screen the geneticin resistance transformants by PCR with split-marker primers (5F: V1-F and Ge-R; 3F: Ge-F and V1-1R). **(C)** Validation of the *VdMcm1* deletion mutants by PCR with primers M1-F and M1-R. The deletion mutants showed one band (the length of geneticin resistance cassette), the wild-type strain showed one different band (the length of *VdMcm1*) whereas the ectopic mutants contained two bands: one for the geneticin resistance cassette, one for *VdMcm1*. **(D)** Semiquantitative RT-PCR was performed to validate the deletion and reintroduction of *VdMcm1*. **(E)** Confirmation of the *VdMcm1* deletion mutant by Southern blot with a *VdMcm1* probe. Genomic DNA of wild-type strain and *VdMcm1* deletion mutant were digested with *Kpn I*.

Figure S2 **Expression analysis of genes related to melanin biosynthesis**. Calculation the transcript levels of five melanin biosynthesis genes with qRT-PCR. The β-tubulin gene was used as a reference gene for the expression analyses. The error bars represent standard deviations. The experiments were performed in triplicate.

Figure S3 **Scanning electron microscopy of smoke tree roots inoculated with conidial suspensions**. Arrows show germinated conidia in the wild-type and complemented strains. The pictures were taken after 24 h of incubation with 10^6^ conidia/ml.

Figure S4 **Validation of mRNA-Seq expression patterns by qRT-PCR**. The qRT-PCR results of the selected genes showed similar expression patterns to those detected by mRNA-Seq. VDAG_08640 and VDAG_08676 are bZIP transcription factor genes; VDAG_03208 is a C_2_H_2_ transcription factor gene; VDAG_00190 is a gene involved in melanin biosynthesis; VDAG_01206 is a pyruvate kinase gene which catalyzes the generation of pyruvate from phosphoenolpyruvate in glycolysis pahtway. The error bars represent standard deviations. The experiments were performed in triplicate.

Figure S5 *****VdMcm1*** regulates genes expression of secondary metabolism gene clusters. (A)** Line graph showing the expression pattern of secondary metabolism gene clusters in Δ*VdMcm1-5* and wild-type strain. Log_2_(FPKM) value was used to draw the picture. **(B)** Heat map showed gene expression profiles of the gene clusters during microsclerotia development (Xiong et al., 2014). CO and GC represent conidia and conidial germination; MS1-MS4 represents four typical stages during the entire process of microsclerotia formation at 60, 72, 96 h, and 14 days.

Table S1 **A list of genes regulated by both ***VdMcm1*** and ***Vta2*****.

Table S2 **A list of secreted protein-encoding genes significantly downregulated in Δ***VdMcm1*****.

Table S3 **All the primers used in this study**.
