# Supplementary figures and images for: MADS-Box Transcription Factor VdMcm1 Regulates Conidiation, Microsclerotia Formation, Pathogenicity, and Secondary Metabolism of Verticillium dahliae
Source: Front Microbiol. 2016 Aug 3;7:1192. doi: 10.3389/fmicb.2016.01192 (PMC4971026; doi:10.3389/fmicb.2016.01192)

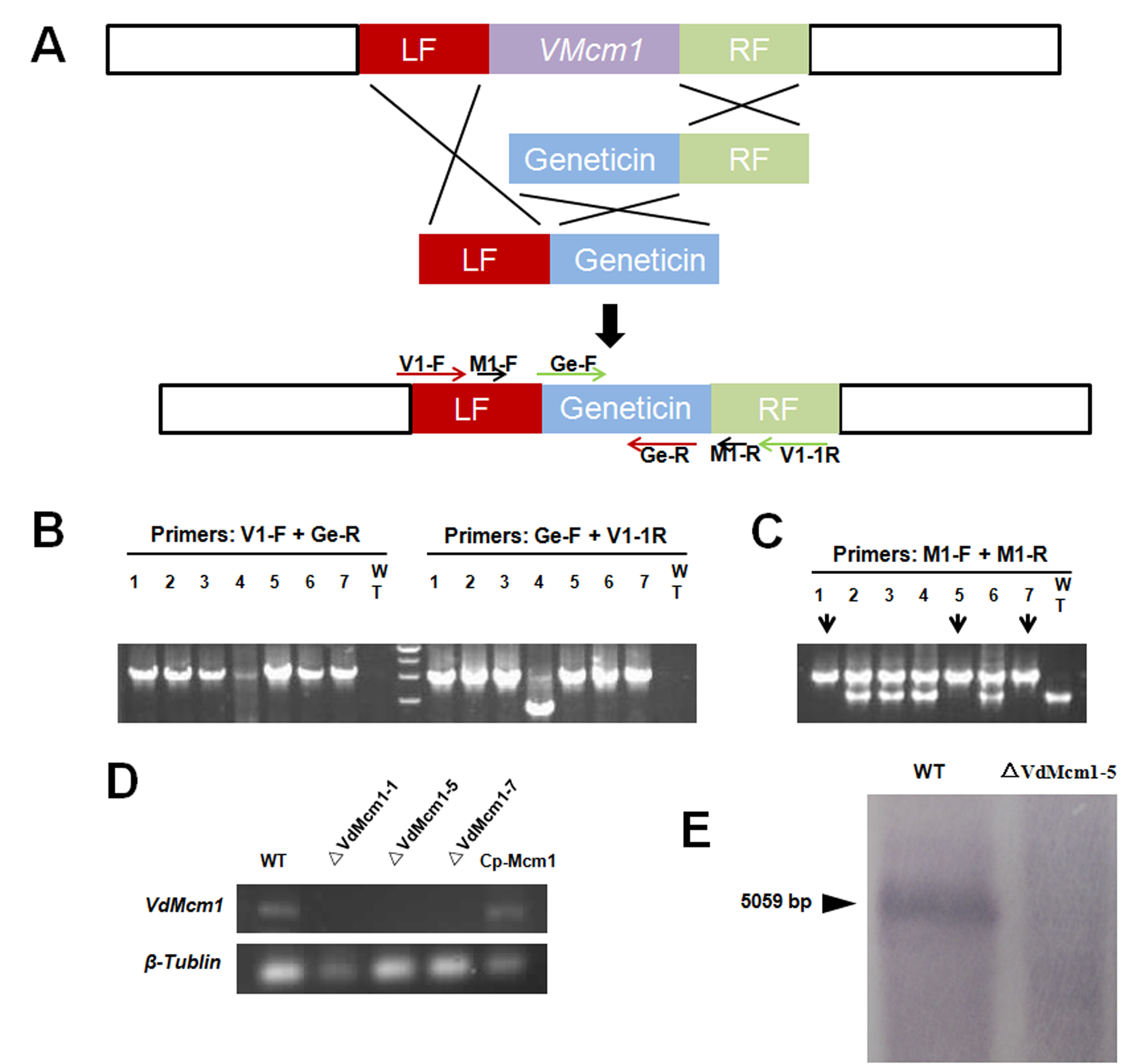

Supplement: Figure S1 — The strategy of gene replacement and validation of gene deletion mutants. (A) Gene replacement strategy for the deletion of VdMcm1. The split-marker method was used. The entire coding sequence of VdMcm1 was replaced with a geneticin resistance cassette. (B) Screen the geneticin resistance transformants by PCR with split-marker primers (5F: V1-F and Ge-R; 3F: Ge-F and V1-1R). (C) Validation of the VdMcm1 deletion mutants by PCR with primers M1-F and M1-R. The deletion mutants showed one band (the length of geneticin resistance cassette), the wild-type strain showed one different band (the length of VdMcm1) whereas the ectopic mutants contained two bands: one for the geneticin resistance cassette, one for VdMcm1. (D) Semiquantitative RT-PCR was performed to validate the deletion and reintroduction of VdMcm1. (E) Confirmation of the VdMcm1 deletion mutant by Southern blot with a VdMcm1 probe. Genomic DNA of wild-type strain and VdMcm1 deletion mutant were digested with Kpn I. [file Image1.TIF]

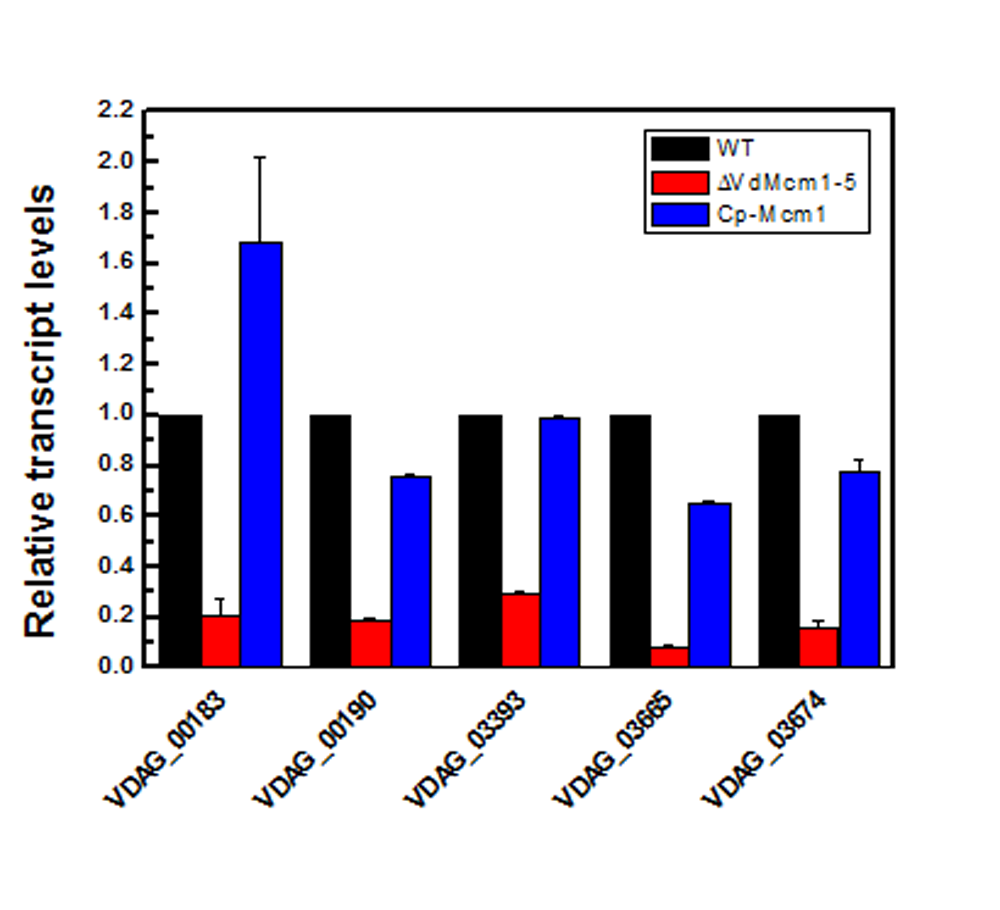

Supplement: Figure S2 — Expression analysis of genes related to melanin biosynthesis. Calculation the transcript levels of five melanin biosynthesis genes with qRT-PCR. The β-tubulin gene was used as a reference gene for the expression analyses. The error bars represent standard deviations. The experiments were performed in triplicate. [file Image2.TIF]

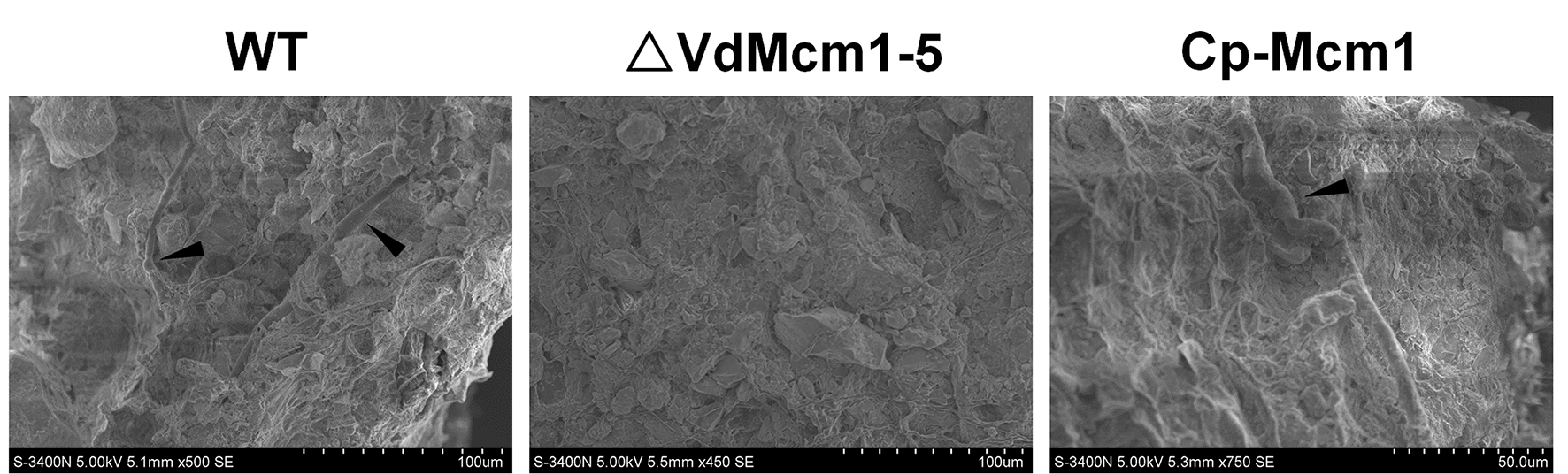

Supplement: Figure S3 — Scanning electron microscopy of smoke tree roots inoculated with conidial suspensions. Arrows show germinated conidia in the wild-type and complemented strains. The pictures were taken after 24 h of incubation with 106 conidia/ml. [file Image3.TIF]

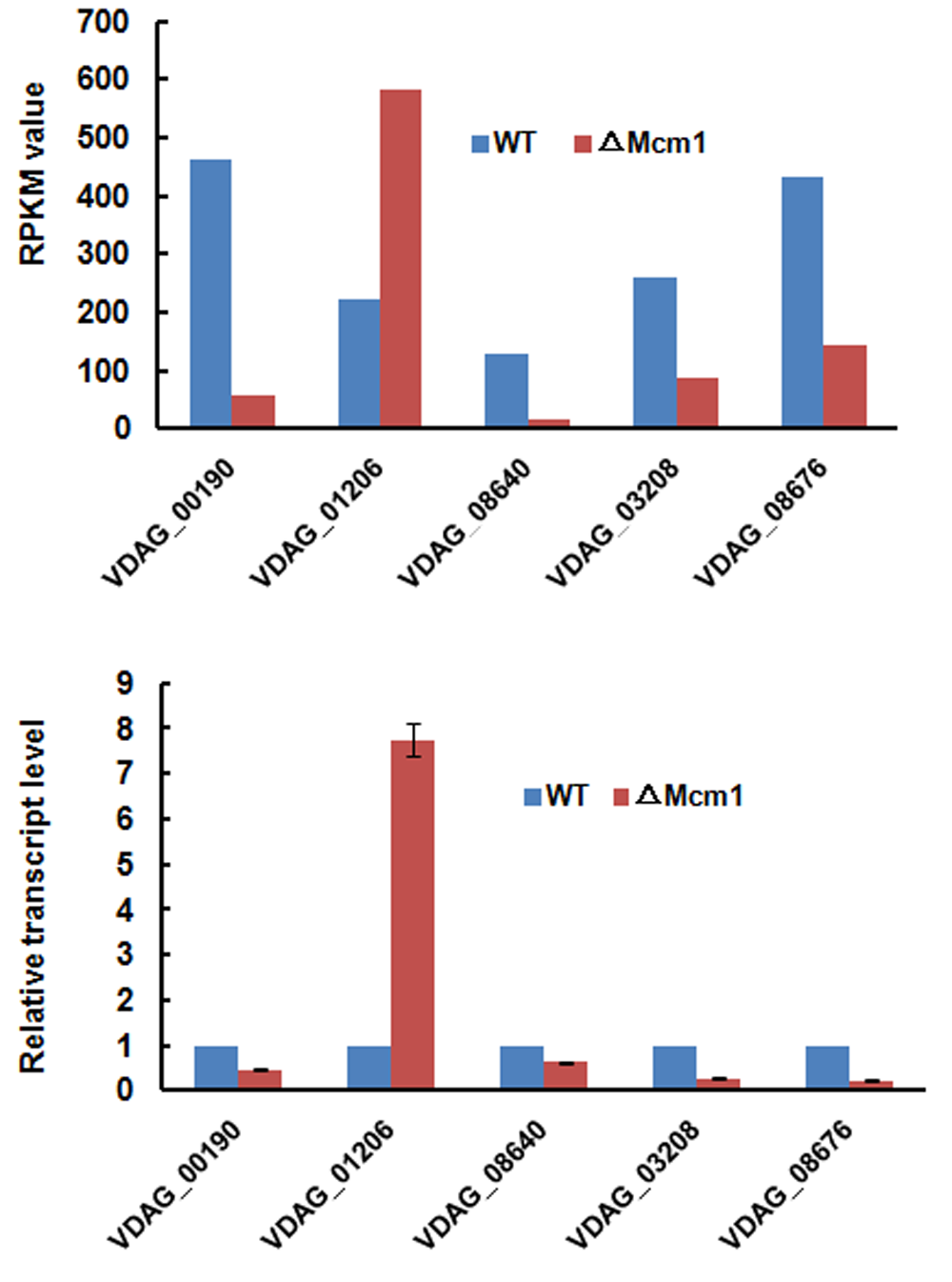

Supplement: Figure S4 — Validation of mRNA-Seq expression patterns by qRT-PCR. The qRT-PCR results of the selected genes showed similar expression patterns to those detected by mRNA-Seq. VDAG_08640 and VDAG_08676 are bZIP transcription factor genes; VDAG_03208 is a C2H2 transcription factor gene; VDAG_00190 is a gene involved in melanin biosynthesis; VDAG_01206 is a pyruvate kinase gene which catalyzes the generation of pyruvate from phosphoenolpyruvate in glycolysis pahtway. The error bars represent standard deviations. The experiments were performed in triplicate. [file Image4.TIF]

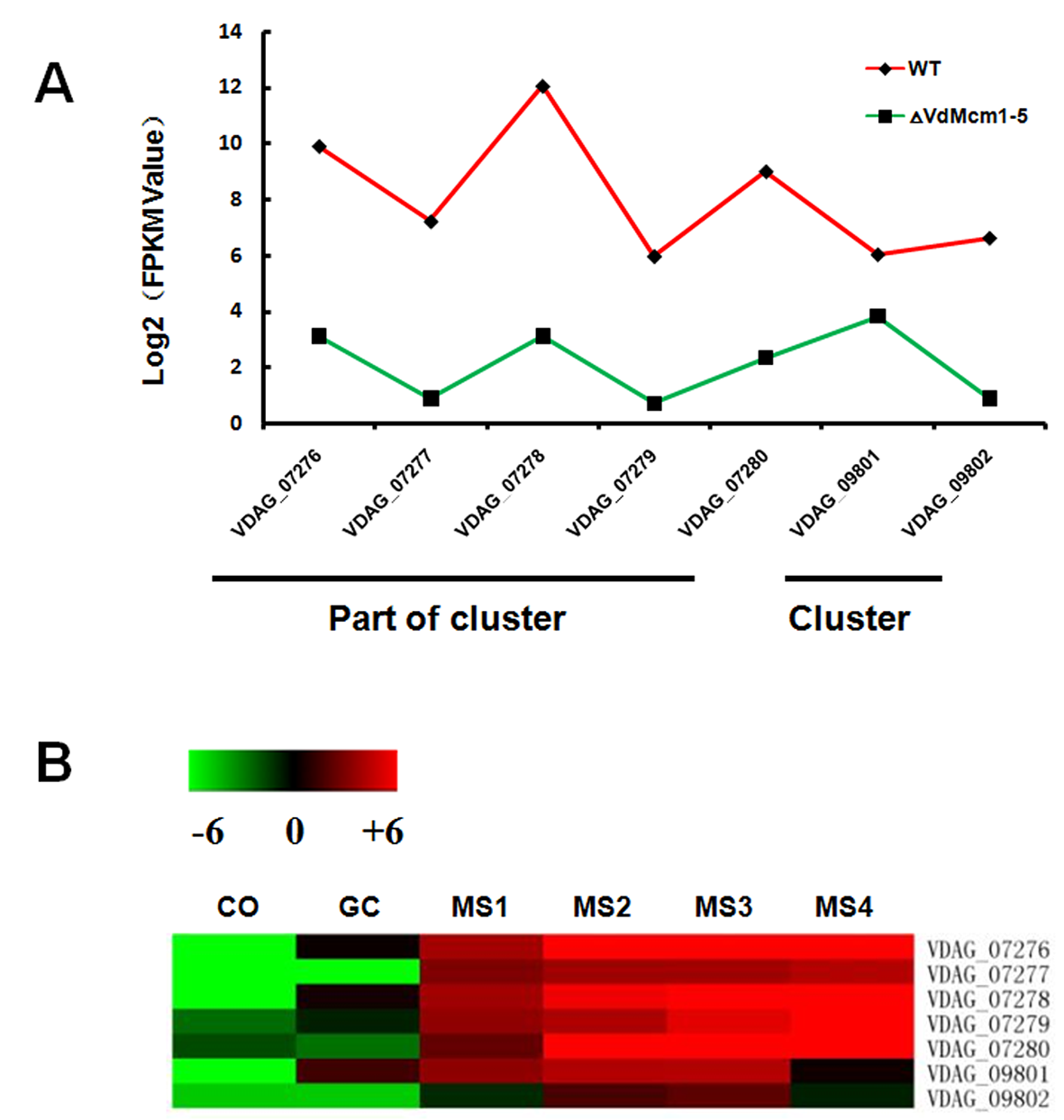

Supplement: Figure S5 — VdMcm1 regulates genes expression of secondary metabolism gene clusters. (A) Line graph showing the expression pattern of secondary metabolism gene clusters in ΔVdMcm1-5 and wild-type strain. Log2(FPKM) value was used to draw the picture. (B) Heat map showed gene expression profiles of the gene clusters during microsclerotia development (Xiong et al., 2014). CO and GC represent conidia and conidial germination; MS1-MS4 represents four typical stages during the entire process of microsclerotia formation at 60, 72, 96 h, and 14 days. [file Image5.TIF]
